# Comparative temporal transcriptome analyses of SARS-CoV-2 delta and omicron variants ex-vivo infection in cat lung explant culture

**DOI:** 10.3389/fcimb.2025.1553464

**Published:** 2025-10-22

**Authors:** Anuradha Panwar, Ashwin Ashok Raut, Sandeep Bhatia, Atul Pateriya, Amod Kumar, Richa Sood, Suneela Saran, Prakriti Sehgal, Arya J. Mohan, Akash Zararia, Anamika Mishra

**Affiliations:** ^1^ Indian Council of Agricultural Research (ICAR)-National Institute of High Security Animal Diseases, Bhopal, India; ^2^ Indian Council of Agricultural Research (ICAR)-National Bureau of Animal Genetic Resources, Haryana, India; ^3^ AASRA, Bhopal, India

**Keywords:** SARS-CoV-2, delta, omicron, domestic cats, ex-vivo, explant, transcriptomic profiling

## Abstract

The rapid evolution of SARS-CoV-2 variants, have posed significant public health challenges due to their heightened transmissibility and varying clinical outcomes. While the impact of these variants in humans has been extensively studied, their effect on domestic animals has not been explored thoroughly. Given the close contact between humans and pets, combined with documented cases of virus transmission from humans to domestic animals and the potential for these animals to act as viral reservoirs, highlights the importance of understanding the molecular dynamics of SARS-CoV-2 infection in them. Consequently, there is an increasing demand for infection models that closely mimic viral behavior in animal tissues. *In vivo* infection of cats or any other non-laboratory animal species is extremely resource intensive, time consuming, require ABSL3 facilities, and animal ethical clearances which are not practically possible for the majority of researchers. In this scenario the ex vivo cultures model can be an excellent alternative. Therefore, in this study, we have utilized an ex vivo lung explant culture model derived from domestic cats to examine the host’s response to Delta and Omicron variants of SARS-CoV-2 in cat lung explant culture. Comprehensive transcriptomic profiling at multiple time points post-infection revealed significant disruptions in genes associated with cell adhesion, structural components, extracellular matrix (ECM) organization and regulation, secreted and growth factors, and immune responses. Our study revealed that the Delta variant triggered early activation of genes associated with tissue damage as early as 12 hours post-infection (hpi). By 24 hpi, both the Delta and Omicron variants showed significant activation of these genes, with the Delta variant linked to the activation of a larger number of tissue damage-related genes. Notably, we identified several hub genes, including *MMP9*, *CCL5*, *MCP-1/CCL2*, *VWF*, *HGF*, *ANGPT1*, *CD34*, *CD68*, *SPP1*, *IGF1*, *CSF1*, and *VCL*, involved in critical signaling pathways such as focal adhesion, PI3K-Akt signaling, and TNF signaling. These hub genes hold potential as valuable biomarkers. This study provides key insights into the pathogenesis of SARS-CoV-2 in cats and highlights the utility of ex vivo lung explant cultures as a platform for studying viral infections.

## Introduction

The global impact of SARS-CoV-2, the causative agent of COVID-19, has been profoundly influenced by the emergence of SARS-CoV-2 variants with enhanced transmissibility, immune evasion, and altered pathogenicity (Chaudhari et al., 2022). Among these, the Delta (B.1.617.2) and Omicron (B.1.1.529) variants have been responsible for driving successive waves of infection, each associated with distinct clinical outcomes (Aleem et al., 2023). This evolving landscape underscores the critical need for validated animal models that can be dynamically adapted to mimic the course of human SARS-CoV-2 infections and associated disease progression. Various animal models, including mice, ferrets, hamsters, mink, and non-human primates, have been widely utilized for studying SARS-CoV-2 (Cool et al., 2022; Gunasekara et al., 2022; Kim et al., 2022; Lee and Lowen, 2021; Muñoz-Fontela et al., 2022). However, these models often present certain limitations. For instance, wild-type mice lack the natural receptors required for viral entry, while transgenic huACE2 mice, ferrets, and hamsters do not fully replicate severe pulmonary disease or acute respiratory distress syndrome (ARDS) (Tamil Selvan et al., 2022). Non-human primates, although closer to humans in terms of disease pathology, face challenges such as limited availability and zoonotic concerns. Similarly, mink require specific pathogen-free environments, complicating their use (Tamil Selvan et al., 2022). In contrast, domestic cats provide a more practical model for studying SARS-CoV-2. Cats can naturally acquire the infection through ACE2 receptor binding, develop respiratory and systemic symptoms during acute infection, and transmit the virus to other cats. These characteristics make them a valuable model for studying the virus’s pathogenesis and transmission dynamics (Zhou et al., 2020; Jiang et al., 2020; Ryan et al., 2021; Sia et al., 2020; Lu et al., 2020; Shuai et al., 2021; Bosco-Lauth et al., 2020; Halfmann et al., 2020; Rudd et al., 2021; Tamil Selvan et al., 2022). The susceptibility of cats to SARS-CoV-2 infection, offer a unique model for studying host-pathogen interactions, as they exhibit key aspects of COVID-19 pathology similar to those seen in humans (Rudd et al., 2021; Tamil Selvan et al., 2022; Saravanan et al., 2022). Previous studies have shown that intratracheal inoculation of wild-type SARS-CoV-2 in domestic cats leads to significant clinical signs—including lethargy, fever, dyspnea, and dry cough—as well as pulmonary lesions such as diffuse alveolar damage and hyaline membrane formation. These pathological features are consistent with the early exudative phase of COVID-19 in humans and are influenced by the distribution of feline ACE2 receptors (Rudd et al., 2021). Furthermore, the transmission of SARS-CoV-2 to domestic cats and other felids from infected owners or caretakers has been well-established (Barrs et al., 2020; Garigliany et al., 2020; Hosie et al., 2021; Klaus et al., 2021). Although direct zoonotic transmission from felids to humans has not been reported, the possibility remains, particularly with the emergence of new viral variants. Investigating the molecular mechanisms of SARS-CoV-2 infection in cats is therefore essential. This can be achieved using transcriptomics and various methodological approaches, such as *in vivo*, *in vitro*, and ex vivo systems. Traditional cell line models, despite their utility for virological studies, fall short in replicating the complexity of intact tissues and fail to account for interactions between different cell types and the extracellular matrix. *In vivo* studies, while providing valuable insights into the systemic and pathological aspects of SARS-CoV-2 infection, are often resource-heavy, time-intensive, and require specialized ABSL3 facilities and ethical approvals, which can limit their widespread use. To address these challenges, we adopted an ex vivo lung explant culture model, which retains the structural integrity and immune microenvironment of the lung tissue, making it a more biologically relevant system for examining host responses to SARS-CoV-2 variants (Gordhan et al., 2024). Although this model overcomes many limitations of *in vitro* studies and avoids the practical constraints of *in vivo* experiments, its application is limited by its finite lifespan and the absence of systemic immune components. Nonetheless, given the emergence of SARS-CoV-2 variants and their documented transmission from infected humans to domestic cats, concerns about cross-species adaptability and potential reservoirs have intensified. While zoonotic transmission from cats back to humans has not yet been reported, the possibility remains, particularly with the ongoing evolution of the virus. These factors underscore the need to investigate the molecular mechanisms of SARS-CoV-2 infection in cats. Therefore, in this study we performed transcriptomic profiling of cat lung explant cultures infected with SARS-CoV-2 Delta and Omicron variants at three distinct time points, enabling the investigation of temporal and variant-specific host responses under physiologically relevant ex vivo conditions. Differential gene expression, functional enrichment, and hub gene network analyses revealed key molecular pathways and regulatory genes associated with viral pathogenesis and immune activation. Hub genes, defined by their extensive interactions and high connectivity within gene networks, often play central roles in coordinating complex biological responses (Yu et al., 2017). To assess the discriminatory potential of these genes, we conducted receiver operating characteristic (ROC) curve analysis using the pROC package in R (Robin et al., 2011). The area under the ROC curve (AUC) served as a quantitative indicator of gene performance across conditions, with values of 0.7–0.8 considered acceptable, 0.8–0.9 excellent, and >0.9 outstanding (Hosmer et al., 2013). Expression profiles of selected hub genes were further validated using real-time PCR. Together, these findings enhance our understanding of SARS-CoV-2 infection dynamics in cats and highlight the relevance of lung explant cultures as a robust ex vivo model for studying respiratory viral infections.

## 2 Materials and methods

### 2.1 Source of experimental tissue

Lung tissue samples were obtained from recently deceased cats (n=3) who have died from non-lung-related causes, such as accidents, fractures, or injuries, ensuring no pre-existing lung pathology in collaboration with AASRA, a Non-Governmental volunteer organization, and the State Veterinary Hospital in Bhopal, India. Following postmortem collection, the tissues were placed on ice in RPMI media supplemented with a 2X concentration of antibiotics (Gentamicin and antimycotic) and transported to the ICAR-National Institute of High Security Animal Diseases (NIHSAD).

### 2.2 Explant culture preparation, virus source, and infection

All experiments were performed in a Biosafety Level 3 (BSL-3) containment facility at the National Institute of High Security Animal Diseases (NIHSAD), Bhopal, India. Lung tissues were collected immediately postmortem and washed twice with RPMI media (Gibco, Thermo Fisher Scientific, USA; Cat. No. 11875093), supplemented with a 2X concentration of antibiotics, including Gentamicin (Thermo Fisher Scientific, USA; Cat. No. 15710064) and Antimycotic Solution (Thermo Fisher Scientific, USA; Cat. No. 15240062). The tissues were then finely chopped into approximately 1 mm³ fragments using sterile curved surgical scissors in a petri dish placed on ice to preserve cellular integrity. The resulting explant suspension was transferred into sterile tubes containing RPMI medium with antibiotics. A portion of this was reserved as a mock (non-infected) control, while the remaining explants were inoculated with SARS-CoV-2 Delta and Omicron variants at a multiplicity of infection (MOI) of 1, using viral stocks previously propagated in Vero E6 cells under BSL-3 conditions. Explants were plated in 6-well culture plates, targeting an approximate cell density of 10^7^ cells per well based on yield estimates from processed tissue. Cultures were incubated at 37 °C in a humidified atmosphere with 5% CO_2_. Mock-infected control samples were harvested at 6 hours post-infection (hpi) to serve as a baseline. Infected explants were collected at 0, 6, 12, and 24 hpi into tubes containing TRIzol™ Reagent (Thermo Fisher Scientific, USA; Cat. No. 15596026).

### 2.3 RNA Isolation, and quantification, and qRT-PCR to check explant culture

Total RNA was extracted from lung explant cultures at mock, 0, 6, 12, and 24 hours post-infection (hpi) using the PureLink™ RNA Mini Kit (Thermo Fisher Scientific, USA; Cat. No. 12183018A), following the manufacturer’s protocol. RNA concentration was determined using the Qubit™ RNA High Sensitivity (HS) Assay Kit (Thermo Fisher Scientific, USA; Cat. No. 2935440).

### 2.4 Checking explants using real time PCR

Quantitative reverse transcription PCR (qRT-PCR) was performed in collected samples (mock, 0hpi, 6hpi, 12hpi, and 24hpi) using VIRALDTECT-II Multiplex Real Time PCR kit for COVID-19 (Genes2Me; Cat. No. G2M020220), containing specific primers and fluorescent probes targeting RdRp, E, and N gene of SARS-CoV-2, following the manufacturer’s instructions.

### 2.5 Data generation

Approximately 500 ng of RNA from mock, 6hpi, 12hpi, and 24hpi samples was used for data generation using Illumina Novaseq 6000 platform. 2 x 150 bases paired-end sequencing was performed to generate approximately 10 Gb of data per sample. RNA quality was assessed prior to sequencing using Nanodrop spectrophotometry (260/280 and 260/230 ratios) and 1% agarose gel electrophoresis to ensure sample integrity. Detailed information on RNA quality control metrics, sample identifiers, sequencing depth, and read alignment percentages is provided in **Supplementary Material 1**.

### 2.6 Data filtration and DEGs identification

Raw sequence data from each sample was subjected to quality control checks using FastQC (Babraham Bioinformatics). Adaptor and low-quality sequences were removed using Cut adapt with the default parameters. Quality filtered reads from mock and infected samples were mapped to *Felis catus* reference genome. The gene counts were obtained using Hisat2 in RSEM (Li and Dewey, 2011). The counts were used for calculating differentially expressed genes (DEGs) by use of R package, DESeq2 (Love et al., 2014). Genes satisfying the criteria of a |log 2 (fold change) | ≥ 1 for upregulated, ≤ -1 for downregulated and the p-value ≤ 0.01 were defined as DEGs. DEGs were identified and visualized using a volcano plot.

### 2.7 Short time-series expression miner analysis

Short Time Series Expression Miner (STEM) software, which clusters genes both by significance and expression patterns (Ernst and Bar-Joseph, 2006) was used to identify longitudinal patterns of gene expression changes throughout infection. We used a number of model profiles set as 10 and a maximum unit change in model profiles between time points set at 1. Gene expression values were transformed to log ratios relative to the expression value at control. Then, each gene was assigned to the filtering criteria of the model profiles, and the correlation coefficient was determined. The boxes in the figures were colored if the profiles were statistically significant and boxes of the same color with high similarity could be analyzed together. Gene Ontology (GO) and Kyoto encyclopedia of genes and genomes (KEGG) pathway analyses of significant profiles were performed using DAVID (Sherman et al., 2022) and a p-value ≤ 0.05 was considered statistically significant.

### 2.8 Construction of a PPI network, module analysis, hub gene identification, and AUC analysis for hub genes

The PPI network was constructed using the Search Tool for the Retrieval of Interacting Genes (STRING; http://string-db.org) (version 12) online database. For omicron variant the PPI network of DEGs from profiles 0 and 9 was constructed, and for delta variant the PPI Network of Profile 0, 5, and 9 was constructed using the STRING database, and interaction with a combined score > 0.4 was considered statistically significant. Then, the results were visualized with CytoScape software v3.10.2. MCODE v2.0.3, a CytoScape plug-in was used for identifying significant modules (MCODE score ≥ 6), the selection criteria were as follows: degree cut-off = 2, MCODE scores ≥ 6, Max depth = 100, node score cut-off = 0.2, and k-score = 2. Further, hub genes were selected using CytoHubba v0.1, which is another plug-in of CytoScape, according to the number of associations with other genes in the PPI network. Five common algorithms (MNC, Degree, Closeness, Radiality, and EPC) were used for evaluating and selecting hub genes. Further, Receiver‐operating characteristic (ROC) analyses was also performed to calculate AUC value of hub genes using R “*pROC*” package (Robin et al., 2011). AUC values between 0.7 and 0.8 are considered acceptable, those between 0.8 and 0.9 are deemed excellent, and values above 0.9 are regarded as outstanding (Hosmer et al., 2013).

### 2.9 Validation using RT-qPCR

To validate the RNA-Seq results, we performed quantitative real-time PCR (qRT-PCR) using the same RNA samples that were utilized for library preparation. qRT-PCR was conducted using the NeoScript One Step qRT-PCR Kit with SYBR Green (Genes2Me; Cat: #OS01) following the manufacturer’s protocol. Each 10 µL reaction contained 5 µL of 2X One Step SYBR Green Mix, 0.5 µL of One Step SYBR Green Enzyme Mix, 0.5 µL each of forward and reverse primers (10 µM), and 3.5 µL of RNA template (15 ng). The thermal cycling conditions were as follows: reverse transcription at 50°C for 3 minutes, initial denaturation at 95°C for 5 minutes, followed by 30–35 cycles of denaturation at 95°C for 10 seconds and annealing/extension at 60°C for 30 seconds. Relative gene expression levels were calculated using the 2^−ΔΔCT method, with GAPDH and ACTB used as internal reference genes. Primers used in this study are listed in **Table 3 of Supplementary Material 1**.

## 3 Results

### 3.1 Confirmation of viral infection in explant culture

Throughout the experiment, the colour of RPMI medium remained unchanged indicating viable, uncontaminated explant culture. Real-time PCR using the VIRALDTECT-II Multiplex kit confirmed viral infection, with Ct values ranging from 24–29 for samples at 0hpi, and between 16–22 for samples collected at 6, 12, and 24hpi after Delta and Omicron variant infection, whereas no amplification was observed in mock (non-infected) samples. Although Ct values decreased over time, the change was modest, suggesting limited replication in our cat lung explant ex vivo model. Details of Ct value obtained across different time points are given in **Supplementary Material 1**.

### 3.2 RNA seq data generation

Raw sequencing data in FASTQ format were obtained for mock, 6 hpi, 12 hpi, and 24 hpi samples, with data sizes ranging from 8 to 17 Gb per sample. Detailed information on RNA quality control metrics, sample identifiers, sequencing depth, and read alignment percentages is provided in **Supplementary Material 1**.

### 3.3 Differentially expressed genes for delta and omicron variant infection at different time points

**Figure 1:**
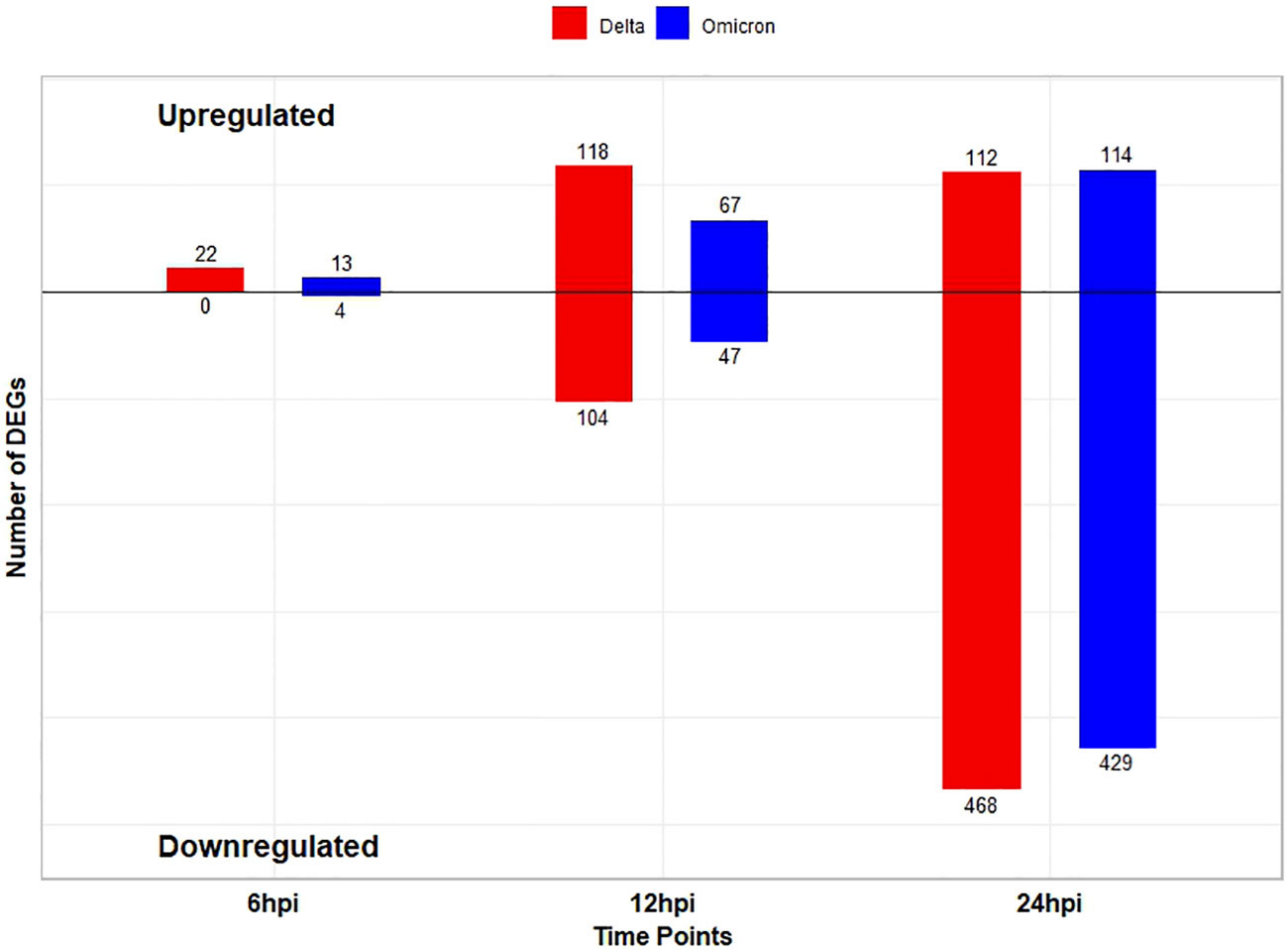
Bar plot showing the number of differentially expressed genes (DEGs) (log2FC ≥ 1 for upregulated and ≤ -1 for downregulated, pvalue ≤ 0.01) at each time point among two variants. Blue bars correspond to the Delta variant, and red bars correspond to the Omicron variant. The numbers above the bars indicate the exact count of DEGs for each condition.

**Figure 2:**
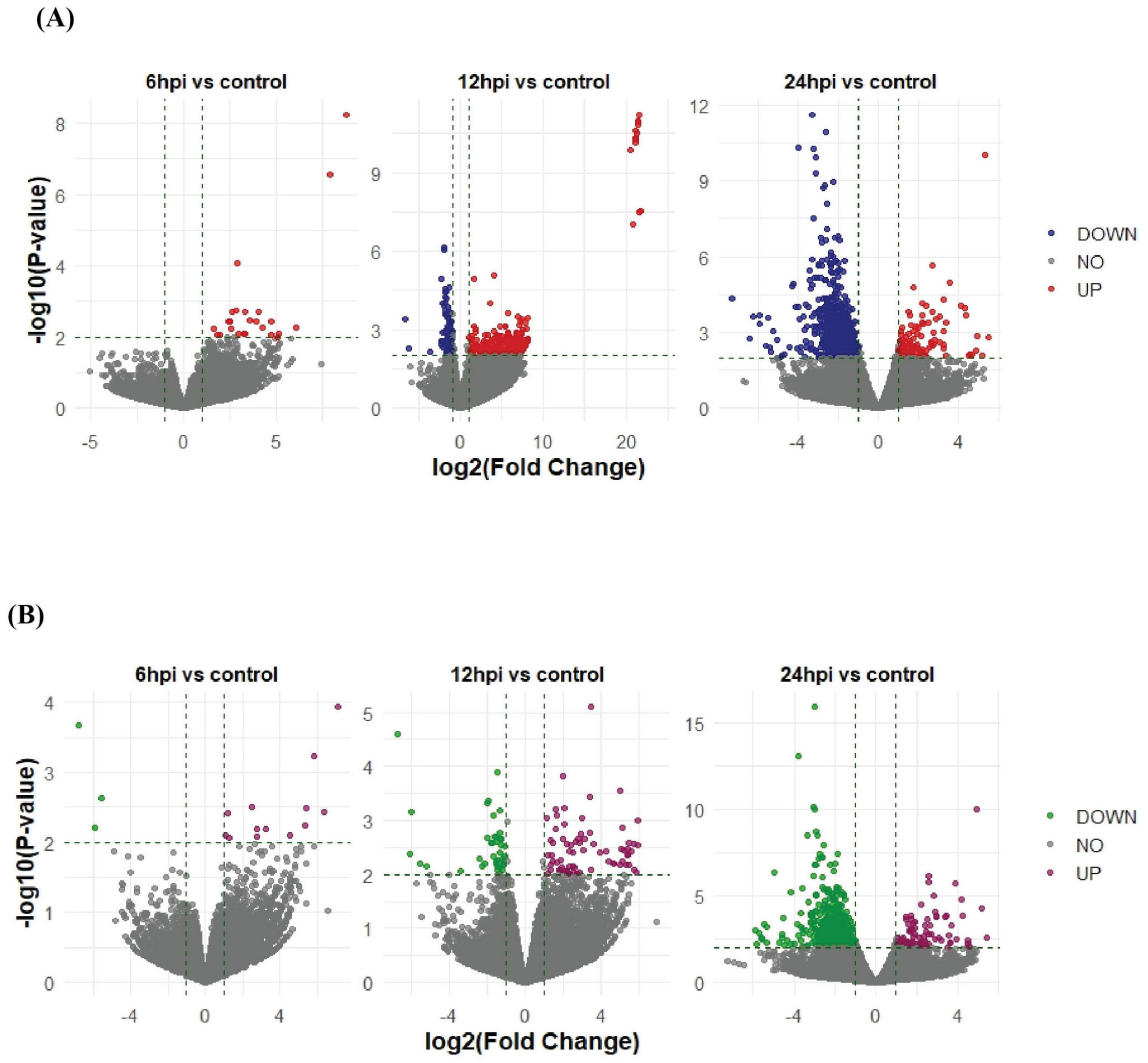
Volcano plots showing differentially expressed genes (DEGs) in cat lung explant cultures infected with the SARS-CoV-2 Delta **(A)** and Omicron **(B)** variants at 6, 12, and 24 hours post-infection (hpi). The x-axis represents the log2(Fold Change) of gene expression, while the y-axis represents the -log10(P-value) of statistical significance.

Following SARS CoV-2 Delta variant infection in cat lung explant culture 22, 222, and 580 DEGs were identified at 6, 12, and 24hpi, respectively. Among these, 22 DEGs were upregulated at 6hpi, with no DEGs downregulated,118 DEGs were upregulated and 104 DEGs were downregulated at 12hpi while 112 DEGs were upregulated and 468 DEGs downregulated at 24hpi (**Figure 1**). Similarly, following Omicron variant infection in cat lung explant culture, 17, 114, and 543 DEGs were identified at 6, 12, and 24 hpi, respectively. Among these, 13 DEGs were upregulated and 4 were downregulated at 6 hpi. At 12 hpi, 67 DEGs were upregulated and 47 were downregulated, while at 24 hpi, 114 DEGs were upregulated and 429 were downregulated (**Figure 1**). **Figures 2A, B** represents the volcano plot after Delta and Omicron variant infection in cat lung explants culture, respectively.

### 3.4 Short time-series expression miner

**Figure 3:**
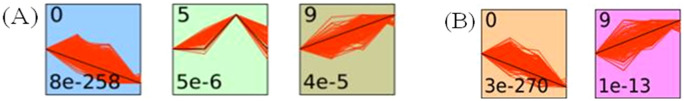
Trend analysis of gene expression in Delta- and Omicron-infected cat lung explant cultures at different time points post-infection using STEM. **(A)** Three significant expression profiles (p-value ≤ 0.01) were identified in Delta-infected cat lung explants. **(B)** Two significant expression profiles (p-value ≤ 0.01) were identified in Omicron-infected cat lung explants. Each profile box represents a model temporal expression pattern generated by STEM. The top-left number indicates the profile ID, and the bottom-left number shows the p-value representing the statistical enrichment of gene assignment to that profile. The black line depicts the overall mean expression trend of all genes assigned to the profile, while the red lines represent the expression trajectories of individual genes, scaled to align with the model profile. Colored backgrounds denote statistically significant profiles, automatically generated by the STEM software.

In this study, to gain insights into the dynamics of gene expression during early disease progression, short time-series expression miner (STEM) software was used to categorize the identified DEGs in the 6hpi vs. Mock, 12hpi vs. Mock, and 24hpi vs. Mock comparisons into a pattern based on 10 profiles. In Delta variant infection, pattern analysis revealed three significant gene expression profiles (**Figure 3A**). Profile 0 was downregulated, Profile 9 was upregulated, and Profile 5 remained temporally stable from 0 to 6 hours post-infection (hpi), then became upregulated from 6 to 12hpi, and finally downregulated from 12 to 24hpi. For Omicron variant infection the pattern analysis results showed that two profiles exhibited significant gene expression patterns (**Figure 3B**), with Profile0 downregulated, and profile 9 upregulated.

#### 3.4.1 GO and KEGG pathway analysis of significant profiles

**Figure 4:**
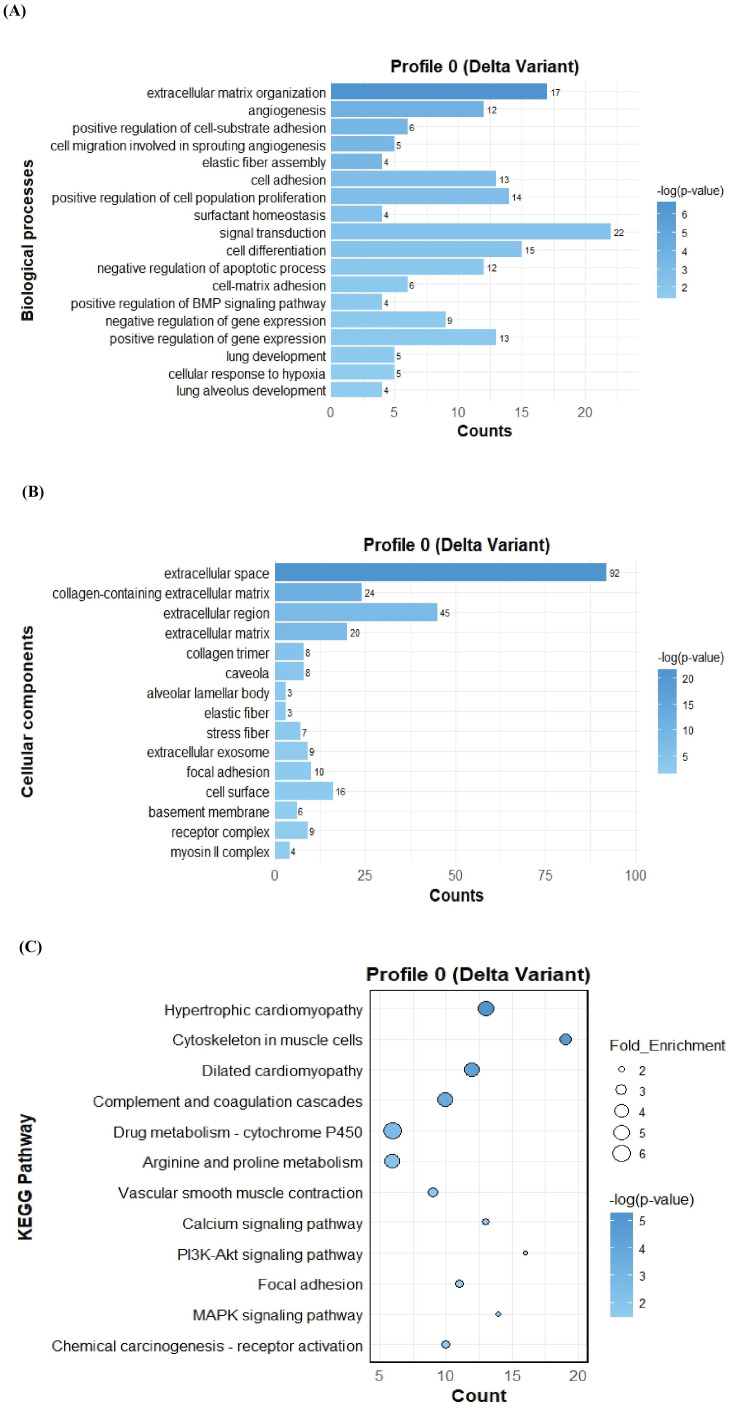
GO term bar graphs representing functional enrichment of genes for **(A)** Biological processes (BP), **(B)** Cellular components (CC), and **(C)** KEGG pathways from profile 0 for Delta variant infection in cat. Horizontal bars represent the number of genes mapping to each GO term, while color intensity represents the –log(p-value) value of the corresponding GO and KEGG terms.

**Figure 5:**
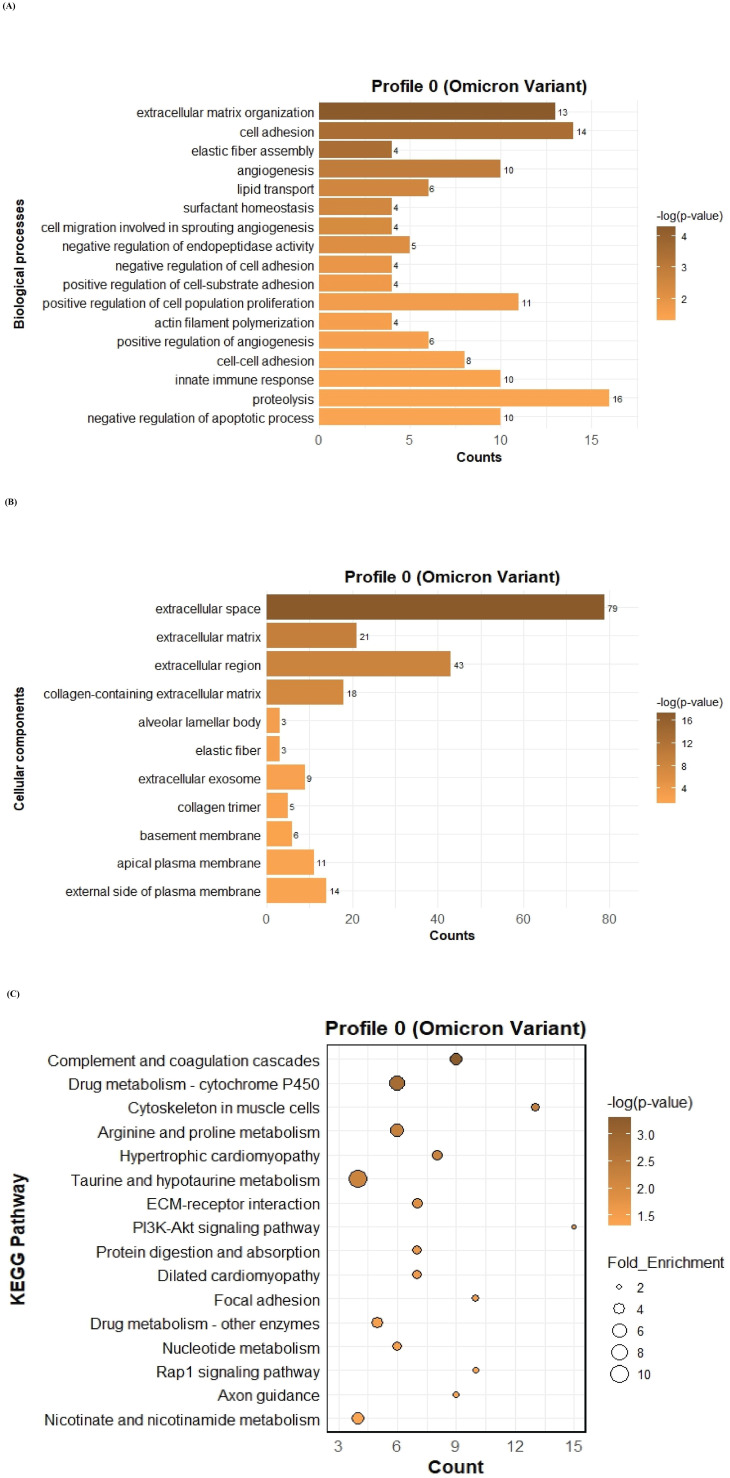
GO term bar graphs representing functional enrichment of genes for **(A)** Biological processes (BP), **(B)** Cellular components (CC), and **(C)** KEGG pathways from profile 0 for Omicron variant infection in cat. Horizontal bars represent the number of genes mapping to each GO term, while color intensity represents the –log(p-value) value of the corresponding GO and KEGG terms.

**Figure 6:**
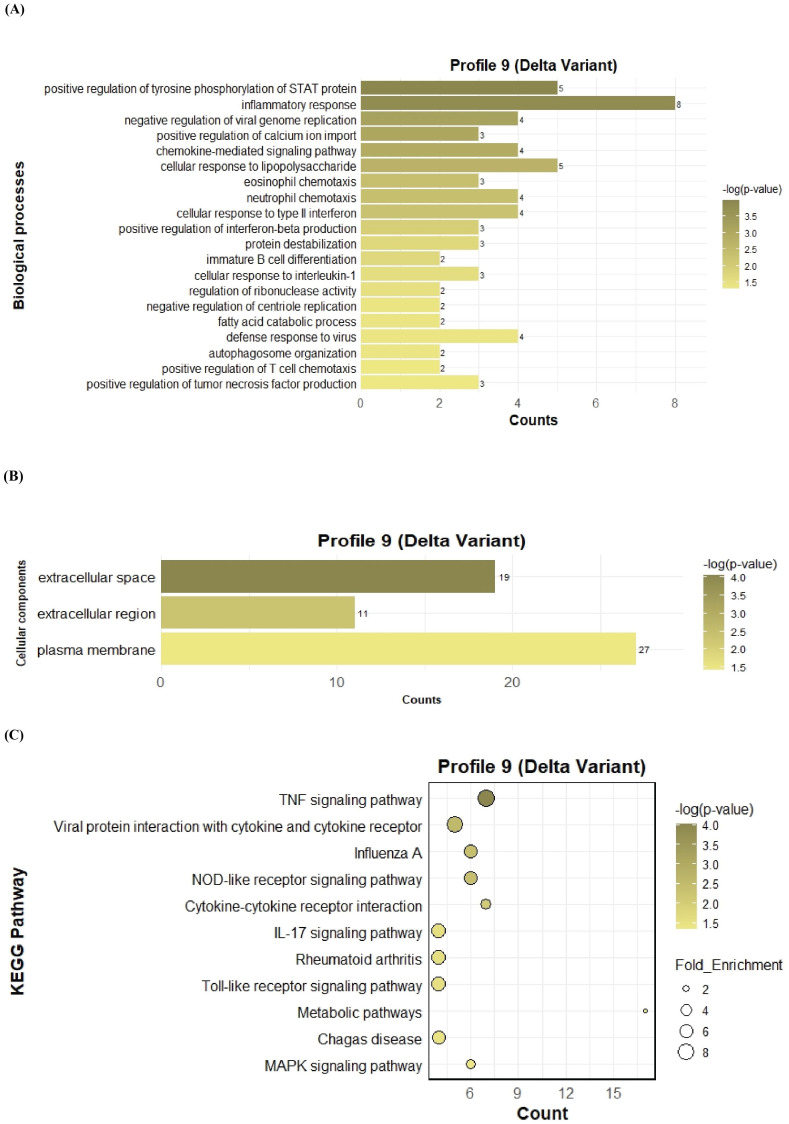
GO term bar graphs representing functional enrichment of genes for **(A)** Biological processes (BP), **(B)** Cellular components (CC), and **(C)** KEGG pathways from profile 9 for Delta variant infection in cat. Horizontal bars represent the number of genes mapping to each GO term, while color intensity represents the –log(p-value) value of the corresponding GO and KEGG terms.

**Figure 7:**
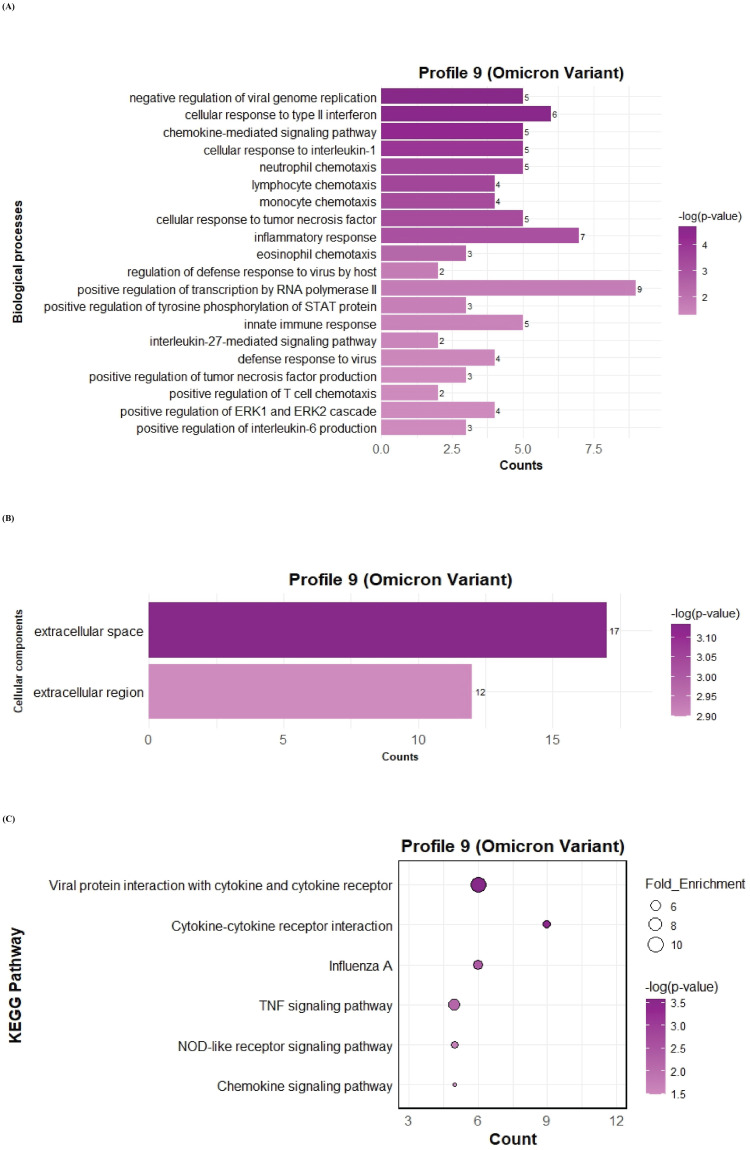
GO term bar graphs representing functional enrichment of genes for **(A)** Biological processes (BP), **(B)** Cellular components (CC), and **(C)** KEGG pathways from profile 9 for Omicron variant infection in cat. Horizontal bars represent the number of genes mapping to each GO term, while color intensity represents the –log(p-value) value of the corresponding GO and KEGG terms.

To further investigate the function of the two significant profiles obtained for Delta and Omicron variants above, we performed Gene Ontology (GO) and KEGG pathway enrichment analyses (p ≤ 0.05) on two major gene expression profiles: Profile 0, comprising significantly downregulated DEGs, and Profile 9, comprising significantly upregulated DEGs. In Profile 0, enrichment of biological processes revealed a range of pathways associated with both Delta and Omicron infections, as shown in **Figures 4A**, **5A**. Pathways commonly enriched in both variants included extracellular matrix organization, angiogenesis, cell adhesion, and surfactant homeostasis. In contrast, some pathways were uniquely enriched in each variant. Signal transduction, fibrinolysis, lung development, and alveolus development were specific to Delta, while proteolysis and innate immune response were uniquely enriched in Omicron infection. These results are provided in detail in **Supplementary Material 2**. Among all enriched pathways, those related to extracellular matrix organization, proteolysis, cell adhesion, and signal transduction involved the largest number of genes, highlighting their critical role in the host response to SARS-CoV-2 infection. The analysis of cellular components associated with downregulated genes in Profile 0 revealed significant enrichment of terms such as extracellular matrix, collagen-containing extracellular matrix, elastic fiber, extracellular region, and basement membrane in both Delta and Omicron infections, as illustrated in **Figures 4B**, **5B**. In comparison, cellular component analysis of upregulated genes in Profile 9 showed that both Delta and Omicron infections were associated with enrichment in the extracellular space and extracellular region, as depicted in **Figures 6B**, **7B**. Key structural components, including the basement membrane and collagen-containing extracellular matrix, were prominently affected, suggesting a loss of tissue architecture and structural integrity during infection. The enrichment of extracellular matrix components points to active tissue remodeling in response to viral damage. Furthermore, changes observed in the plasma membrane and extracellular region imply alterations in cell surface interactions that are essential for immune coordination and intercellular communication. KEGG pathway analysis for Profile 0 indicated that complement and coagulation cascades, focal adhesion, and the PI3K-Akt signaling pathway were enriched in both Delta and Omicron infections, as shown in **Figures 4C**, **5C**. For Profile 9, the biological processes enriched in Delta-infected tissue involved key inflammatory mechanisms, including the inflammatory response, chemokine-mediated signaling, cellular response to interleukin one, and cellular response to type two interferon, which are presented in **Figure 6A**. In Omicron-infected tissue, the upregulated genes in Profile 9 were enriched in processes such as inflammatory response, positive regulation of the ERK one and ERK two cascade, IL-27 mediated signaling, negative regulation of viral genome replication, among others, as shown in **Figure 7A**. KEGG pathway analysis of Profile 9 revealed that both Delta and Omicron infections showed enrichment in pathways such as viral protein interaction with cytokine and cytokine receptors, cytokine-cytokine receptor interaction, influenza A, NOD-like receptor signaling, TNF signaling, and chemokine signaling, as illustrated in **Figures 6C**, **7C**. However, several pathways, including metabolic pathways, interleukin seventeen signaling, and mitogen-activated protein kinase signaling, were uniquely enriched in Delta infection. Taken together, the enrichment patterns indicate that genes involved in focal adhesion and extracellular matrix receptor interaction pathways were consistently downregulated, while genes associated with inflammatory responses and cytokine signaling were consistently upregulated in SARS-CoV-2 infected tissue. This relationship between immune activation and extracellular matrix dysregulation suggests that inflammation triggered by the virus may drive degradation and remodeling of the lung tissue microenvironment. A comprehensive list of all enriched Gene Ontology and KEGG pathways is available in **Supplementary Material 2**.

#### 3.4.2 Screening of genes in profile 0 following delta and omicron variant infection

**Figure 8:**
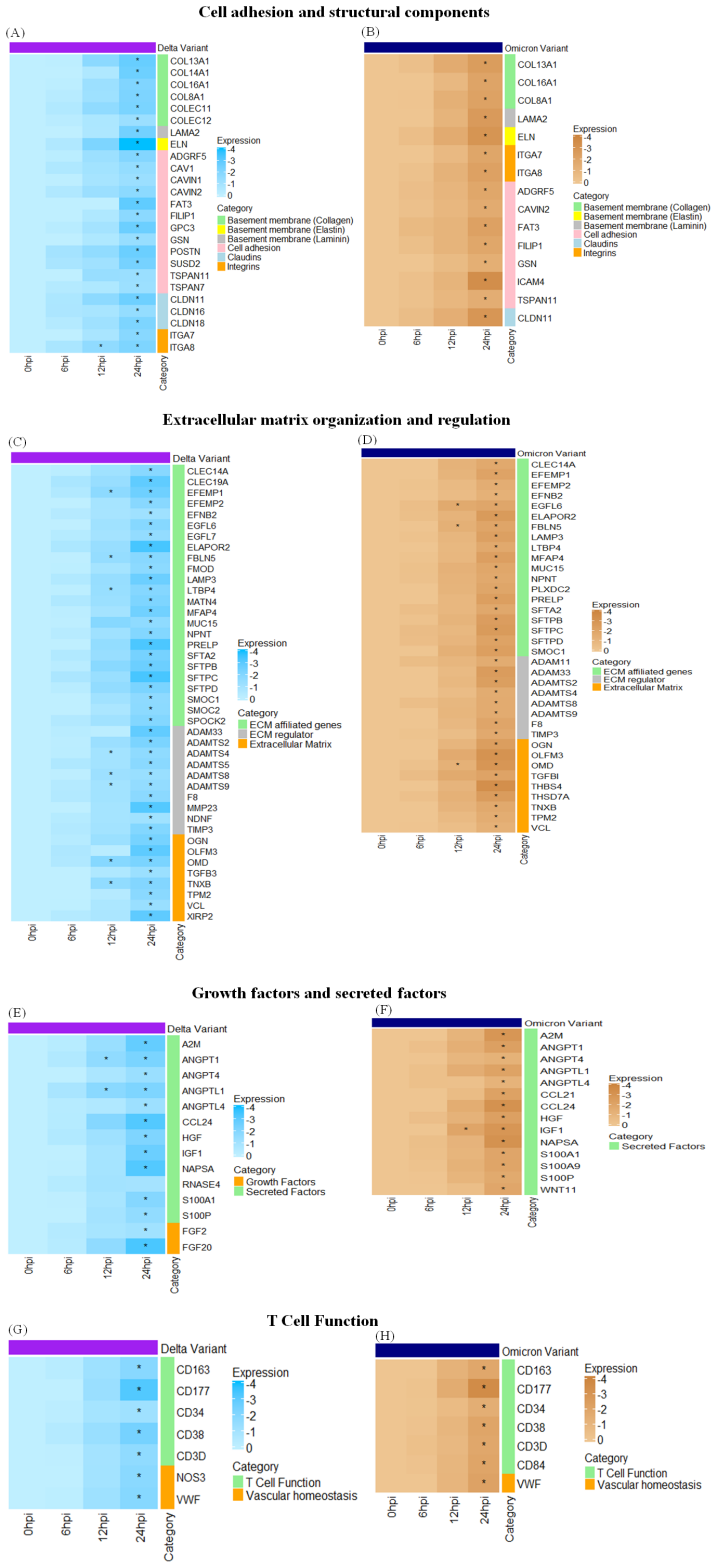
Heatmaps displaying the differential expression of genes across key functional categories like cell adhesion and structural components **(A, B)**, extracellular matrix organization and regulation **(C, D)**, growth factors and secreted factors **(E, F)**, T Cell function **(G, H)** in cat lung explant cultures infected with SARS-CoV-2 Delta and Omicron variant infection, respectively. These genes represent downregulated DEGs identified in Profile 0. Genes with statistically significant expression changes (p-value ≤ 0.01) are indicated with asterisks (*).

Transcriptomic analysis of Profile 0 revealed significant downregulation of genes related to cell adhesion and structural integrity, ECM regulation and organization, secreted and growth factors as well as several immune response genes for both delta and omicron variants. This disruption is observed in both Delta and Omicron variant infections, with distinct differences in the magnitude and specificity of these changes between the two variants. Genes involved in cell adhesion and structural components, including laminins (*LAMA2*), elastin (*ELN*), and collagens (*COL16A1*, *COL13A1*, *COL8A1*) were significantly downregulated at 24 hours post-infection (hpi) in both variants. However, *COL14A1*, *COLEC11*, and *COLEC12* were uniquely downregulated following Delta infection at 24 hpi. Notably, the suppression of *ELN* was more pronounced in Delta-infected tissues, suggesting a greater impairment in lung elasticity compared to Omicron. Among tight junction-associated genes, *CLDN11* was downregulated in both infections at 24 hpi, whereas *CLDN16* and *CLDN18* were specifically downregulated following Delta infection. Several integrins such as *IGF1*, *ITGA8*, and *ITGA7* were consistently downregulated in response to both variants. Additionally, genes related to cell adhesion such as *ADGRF5, CAVIN2*, *FAT3*, *FILIP1*, *GSN*, and *TSPAN11* were downregulated in both the variants at 24hpi whereas *CAV1*, *CAVIN1*, *GPC3*, *POSTN*, *SUSD2*, and *TSPAN7* were downregulated only after Delta variant infection at 24 hpi. A broad downregulation of ECM-affiliated genes was observed, including *CLEC14A*, *EFEMP2*, *EFNB2*, *MUC15*, *LAMP3*, and *PRELP*, all significantly downregulated in both variants at 24 hpi. *EFEMP1* and *LTBP4* were additionally downregulated at 12 hpi and 24 hpi following Delta infection, while *EGFL6* and *FBLN5* showed consistent downregulation at both time points in Omicron infection. Surfactant-associated genes (*SFTPB*, *SFTPC*, *SFTPD*, *SFTA2*) also demonstrated marked downregulation during both variant infections. Several genes involved in ECM regulation, including *ADAMTS2*, *ADAM33*, *ADAMTS5*, *ADAMTS8*, and *ADAMTS9*, were significantly downregulated. Among these, *ADAMTS4*, *ADAMTS8*, and *ADAMTS9* were downregulated at both 12 and 24 hpi in Delta-infected lungs, indicating persistent ECM disruption and likely impairment in tissue repair and remodeling processes. Several secreted and growth factor related genes also showed widespread downregulation. Commonly affected genes across both variants at 24 hpi included *ANGPT1*, *ANGPT4*, *ANGPTL1*, *ANGPTL4*, *CCL24*, *HGF*, *IGF1*, *NAPSA*, *S100A1*, and *S100P*. Notably, *ANGPT1* and *ANGPTL1* were suppressed at both 12 and 24 hpi specifically in Delta infection. Additionally, growth factors such as *FGF2* and *FGF20* were downregulated only in Delta infection and remained unaffected in Omicron. These findings underscore variant-specific differences in the regulation of key factors involved in lung function and repair. Lastly, other immune-related genes such as *VWF*, *CD34*, *CD38*, *CD3D*, *CD84*, *CD163*, and *CD177* were downregulated in both variants at 24 hpi, further emphasizing the extensive molecular alterations triggered by SARS-CoV-2 in feline lung tissue. **Figure 8** represents the heatmap displaying the differential expression of genes across key functional categories in cat lung explant cultures infected with SARS-CoV-2 Delta and Omicron variants, including (A) cell adhesion and structural components, (B) extracellular matrix (ECM) organization and regulation, (C) growth factors and secreted factors, and (D) few others. These genes represent downregulated DEGs identified in Profile 0. Genes with statistically significant expression changes (p-value ≤ 0.01) are indicated with asterisks (*).

#### 3.4.3 Screening of genes in profile 9 following delta and omicron variant infection

**Figure 9:**
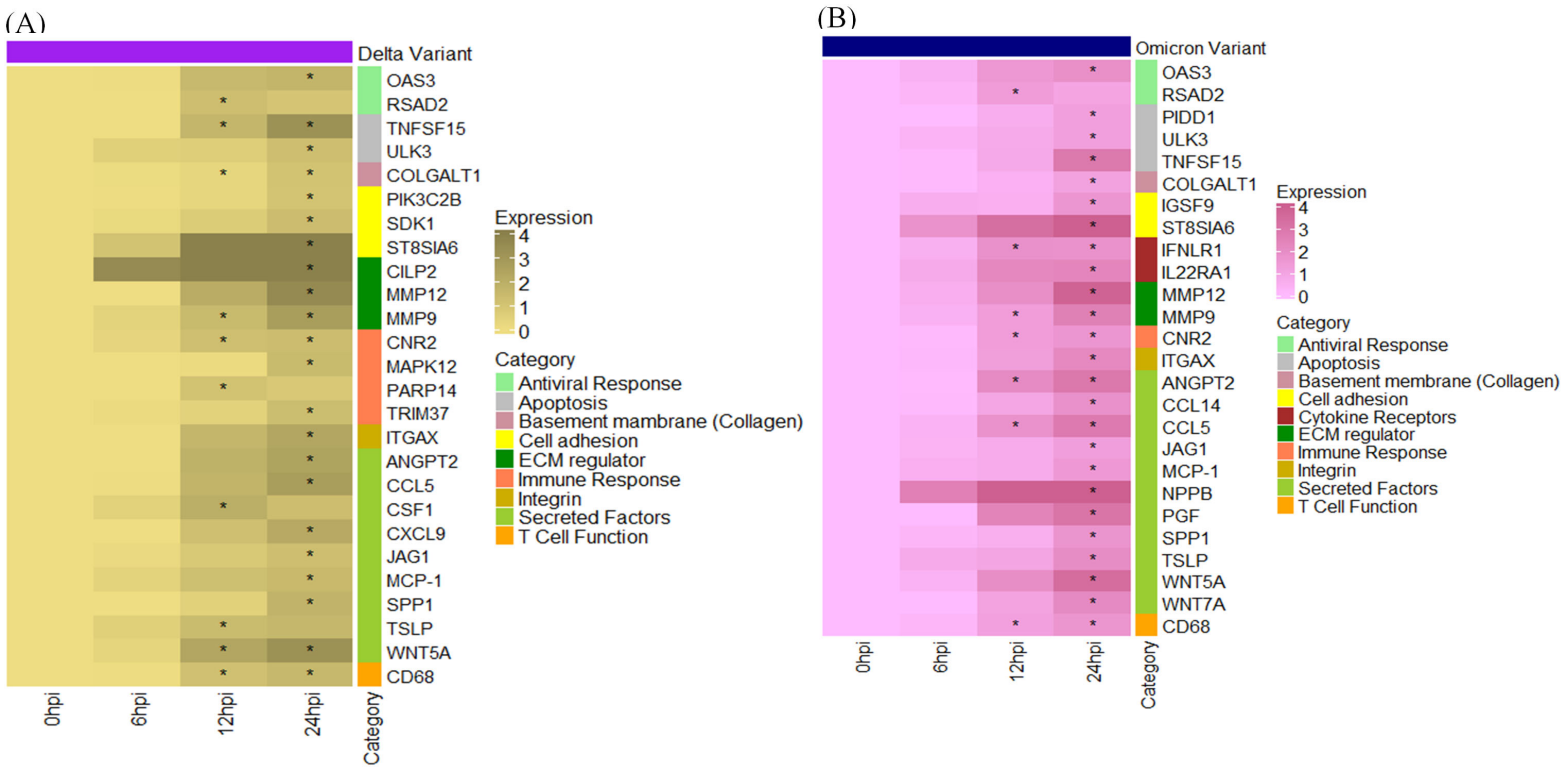
Heatmap displaying the differential expression of genes involved in antiviral response, immune Signaling, and tissue remodeling following SARS-CoV-2 **(A)** Delta and **(B)** Omicron variant infection in cat lung explant culture. These genes represent upregulated DEGs identified in Profile 9. Genes with significant p-value ≤ 0.01 are marked with asterisks (*).

In Profile 9, we observed a significant upregulation of genes involved in secretory and immune responses, with moderate upregulation of genes related to antiviral defense, cell adhesion, integrins, and extracellular matrix (ECM) regulation. **Figure 9** represents the heatmap displaying the differential expression of genes involved in antiviral response, immune Signaling, and tissue remodeling following SARS-CoV-2 (A) Delta and (B) Omicron variant infection in cat lung explant culture. These genes represent upregulated DEGs identified in Profile 9. Genes with significant p-value ≤ 0.01 are marked with asterisks (*). Among the secreted and inflammatory mediators, *ANGPT2, CCL5, JAG1, SPP1*, and *MCP-1* were strongly upregulated in both Delta and Omicron infections, indicating activation of cytokine signaling and immune cell recruitment pathways. Genes associated with ECM remodeling and structural integrity, including *COLGALT1, ITGAX, MMP9*, and *MMP12*, were also elevated. Notably, *COLGALT1* showed consistent upregulation at 12 and 24 hours post-infection (hpi) following Delta infection. *MMP9* was upregulated in both Delta and Omicron infections at 12 and 24 hpi, while *ITGAX* and *MMP12* were specifically elevated at 24 hpi in both variants. In the context of antiviral responses, *OAS3* and *RSAD2* were upregulated predominantly at 24 hpi, reflecting a host response to viral replication. This suggests a late induction of antiviral defenses, which may not be sufficient to counter high viral load early during infection.

### 3.5 PPI network construction of significant profiles and molecular complex detection cluster modules identification

**Figure 10:**
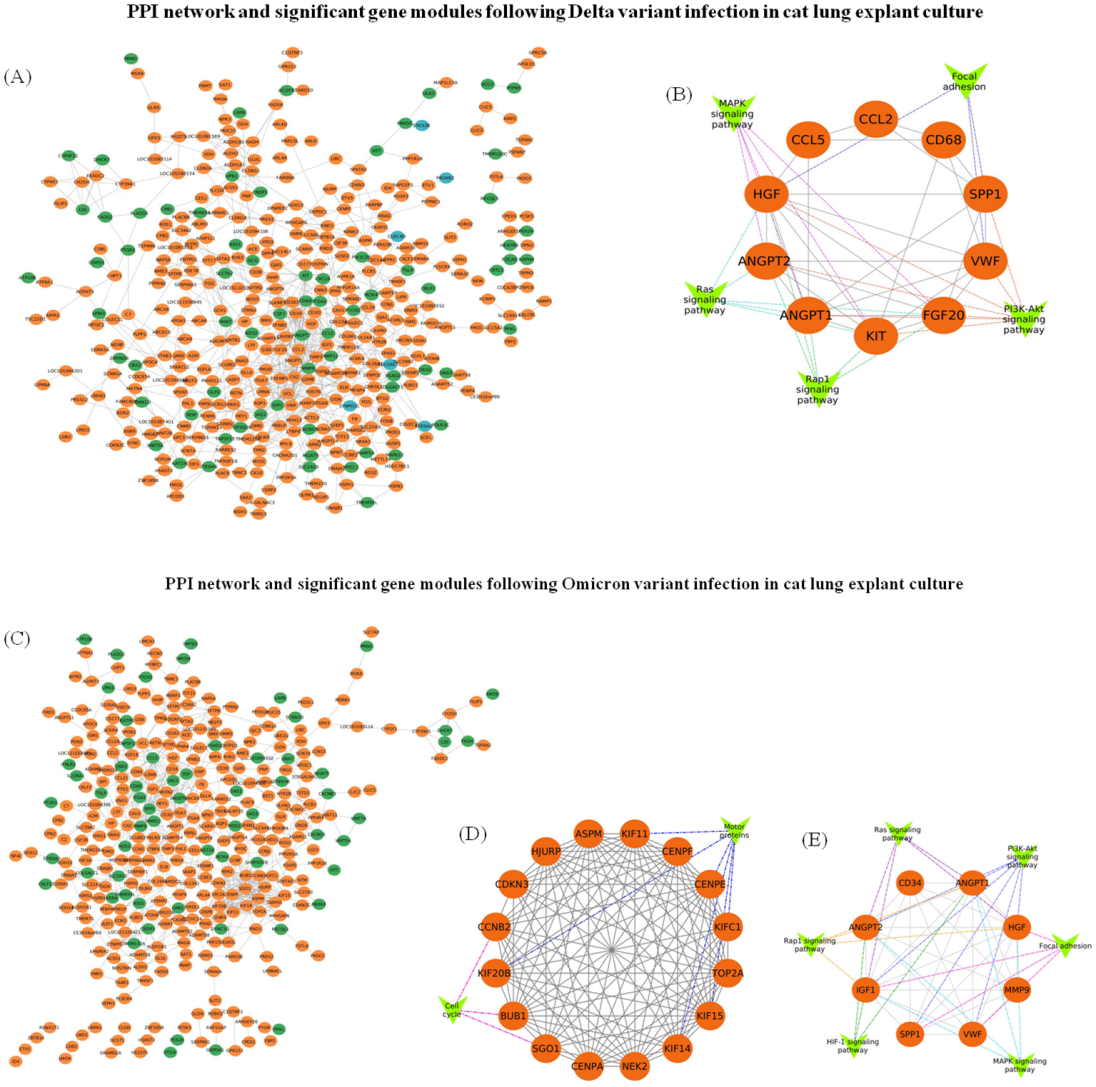
PPI network construction and module analysis following SARS-CoV-2 Delta and Omicron variant infection in cat lung explant culture. **(A)** The PPI network of genes in Profile 0 (orange), Profile 5 (blue), and Profile 9 (green) was constructed using Cytoscape following Delta variant infection. **(B)** A significant gene module (module score ≥ 6) along with associated gene groups (V-shaped nodes) identified from the Delta variant infection. **(C)** The PPI network of genes in Profile 0 (orange) and Profile 9 (green) constructed using Cytoscape following Omicron variant infection. **(D, E)** represent significant gene modules (module score ≥ 6) with gene groups attached (V-shaped nodes) identified following Omicron variant infection.

The PPI network of genes for significant profiles was constructed separately for Delta (**Figure 10A**) and Omicron (**Figure 10C**) variant infection using STRING and the network was visualized by Cytoscape, and the most significant module was identified using Cytoscape plug-in MCODE. MCODE score of ≥ 6 was set as a threshold. For Delta variant infection the analysis revealed one significant module, cluster 1 with MCODE score = 6.2, having 10 nodes and 28 edges (**Figure 10B**). KEGG analysis showed that the genes in this cluster were related to focal adhesion, MAPK signalling pathway, PI3K-Akt signalling pathway, Rap1 signaling pathway, and Ras signalling pathway. For Omicron variant two significant modules were identified with MCODE score of ≥ 6. The first module (Cluster 1) had an MCODE score of 14.4, consisting of 16 nodes and 108 edges (**Figure 10D**). KEGG pathway analysis for this cluster showed that the genes were associated with pathways such as cell cycle, and motor proteins. While the second module (Cluster 2) had an MCODE score of 7.7, containing 8 nodes and 27 edges (**Figure 10E**), KEGG pathway analysis showed that genes in this cluster were enriched in pathways related to HIF-1 signaling pathway, focal adhesion, MAPK signalling pathway, PI3K-Akt signalling pathway, Rap1 signaling pathway, and Ras signalling pathway.

### 3.6 Selection and analysis of hub genes from genes in profile 0 and profile 9

**Figure 11:**
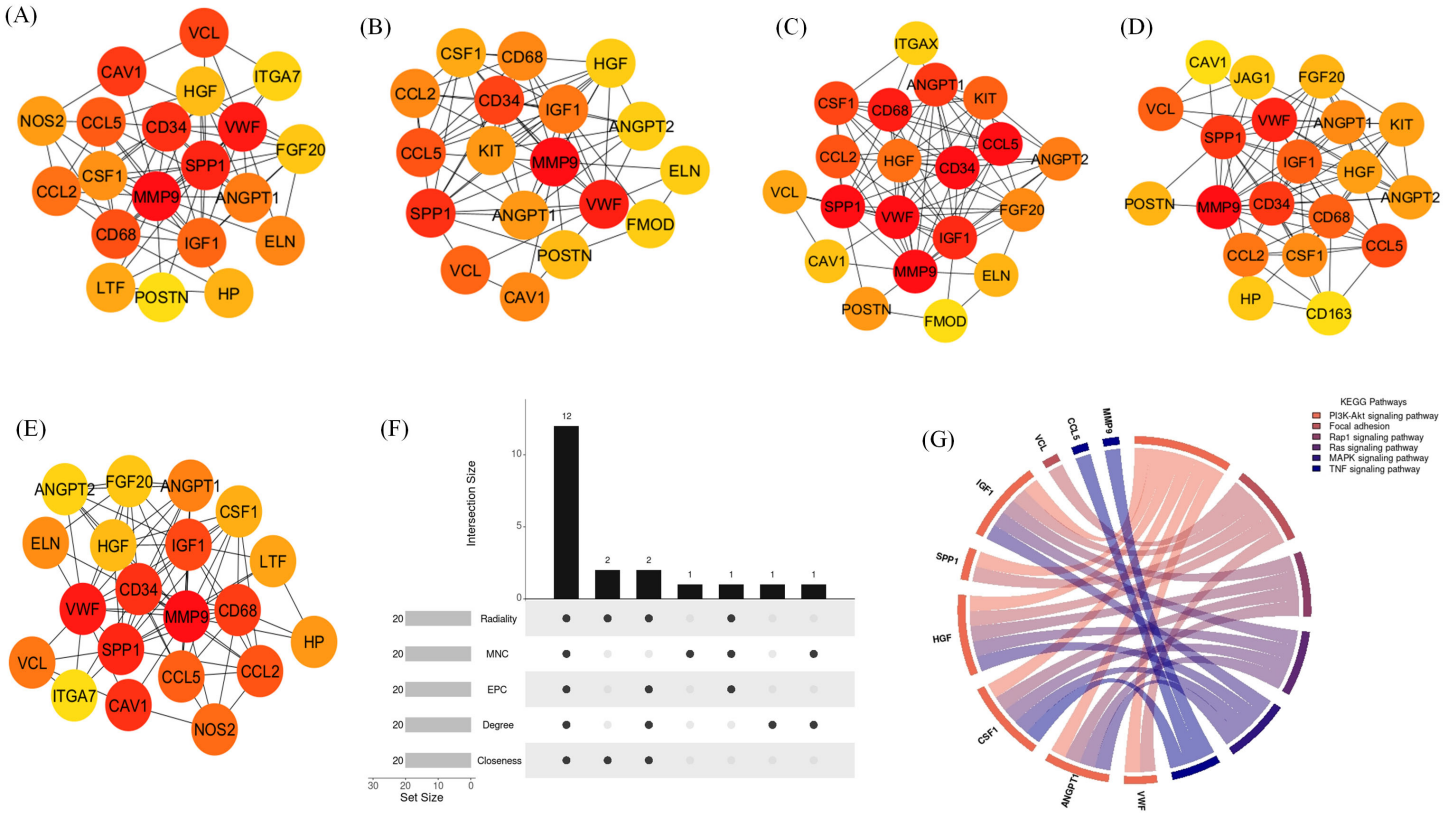
Identification of hub genes and associated KEGG pathways following Delta variant infection in cat lung explant culture using multiple algorithms. **(A–E)** display hub genes identified by five distinct algorithms: Closeness, Degree, EPC, MNC, and Radiality, **(F)** shows an UpSet diagram depicting the overlap across five algorithms, revealing 12 common hub genes, **(G)** represents the KEGG pathways enriched in these hub genes, highlighting their biological significance.

**Figure 12:**
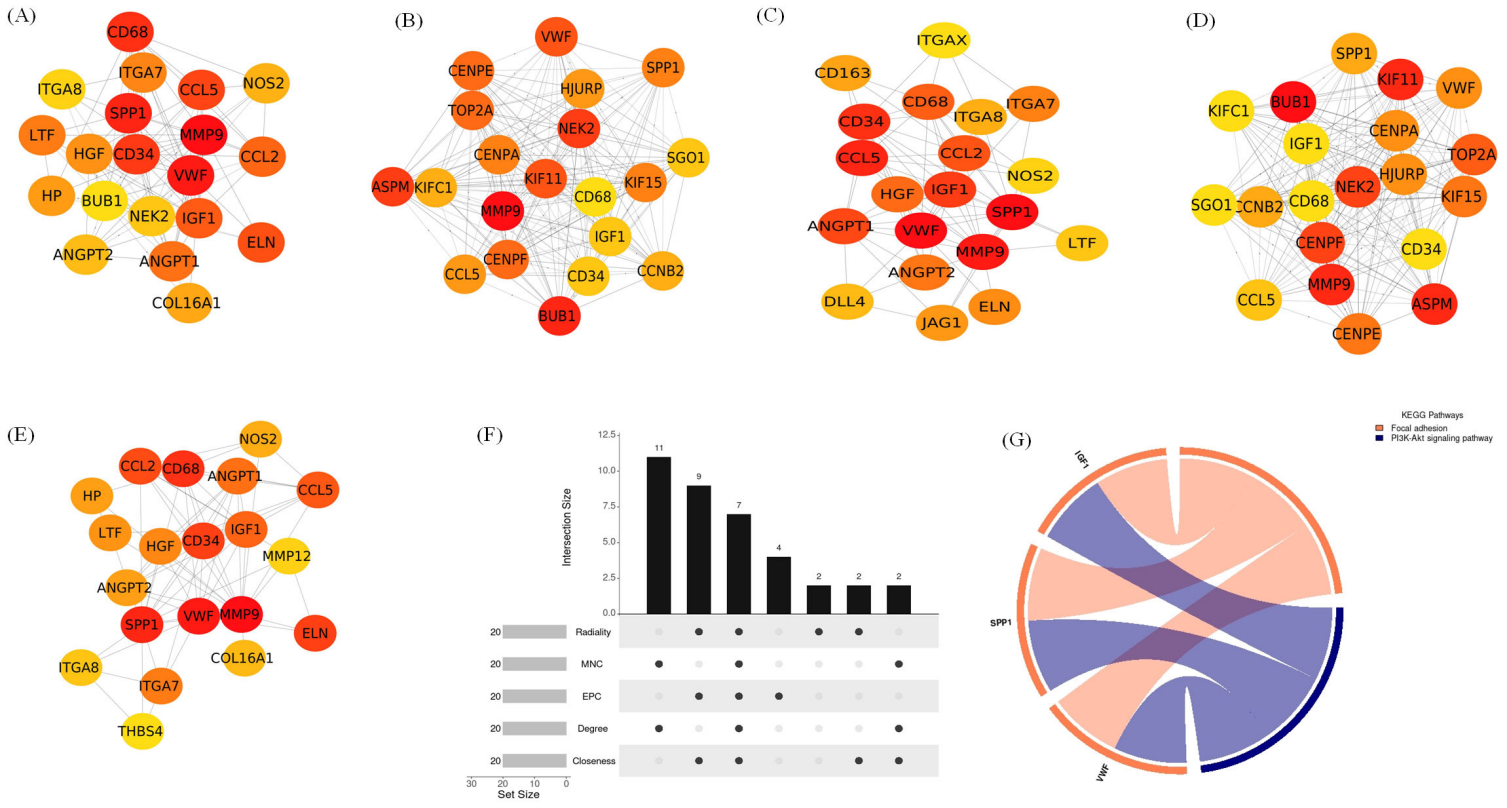
Identification of hub genes and associated KEGG pathways following Omicron variant infection in cat lung explant culture using multiple algorithms. **(A–E)** display hub genes identified by five distinct algorithms: Closeness, Degree, EPC, MNC, and Radiality, **(F)** shows an UpSet diagram depicting the overlap across five algorithms, revealing 7 common hub genes, **(G)** represents the KEGG pathways enriched in these hub genes, highlighting their biological significance.

**Figure 13:**
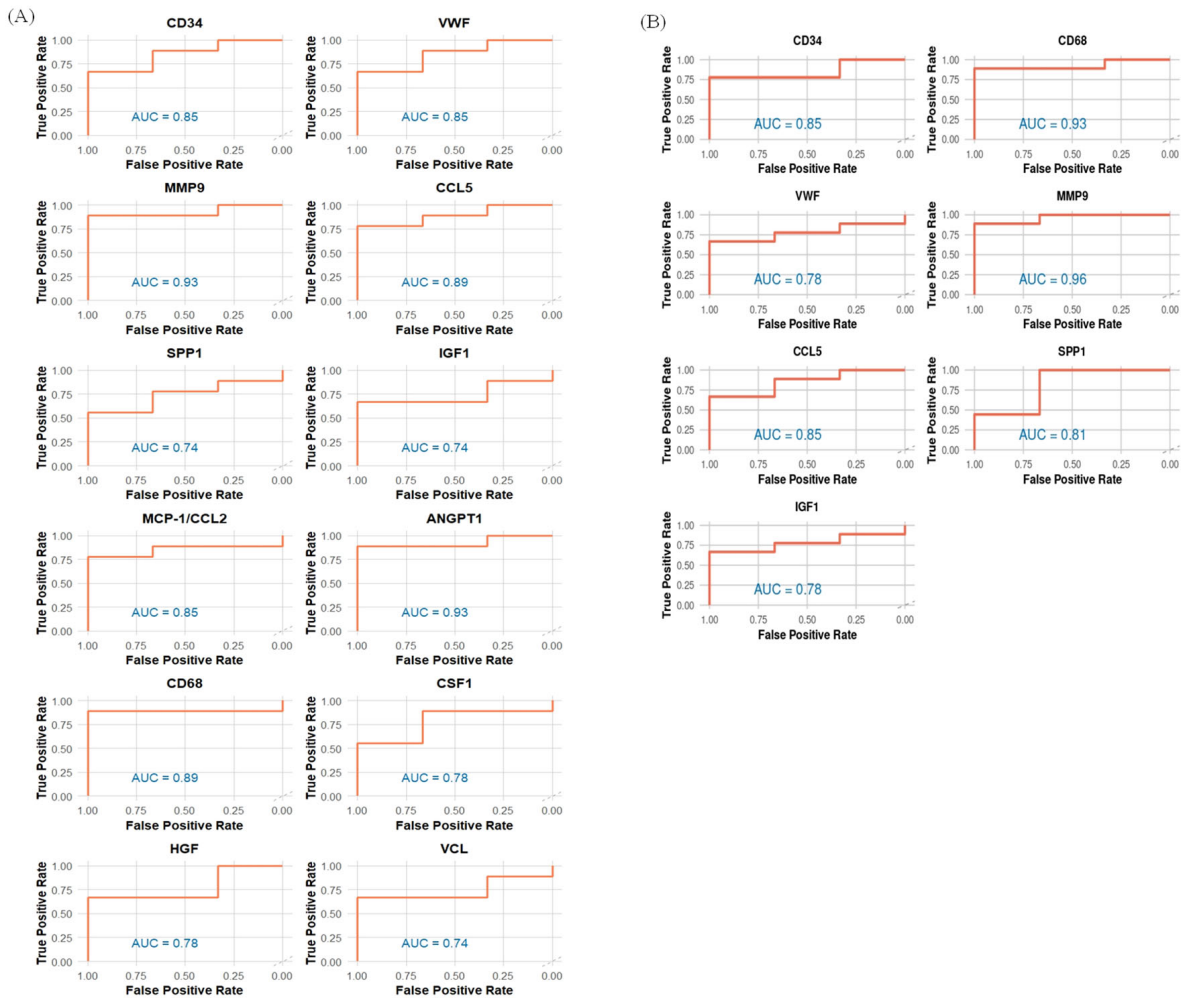
AUC values for identified hub genes for **(A)** Delta and **(B)** Omicron variant infection in cat lung explant culture.

The top 20 hub genes were identified using five distinct algorithms within the CytoHubba plug-in, analyzed separately for the Delta variant (**Figures 11A–E**) and the Omicron variant (**Figures 12A–E**). Each algorithm generated a ranked list of hub genes based on network topology features. To identify the most consistently central genes across methods, we used an UpSet diagram to visualize the overlap among the five ranking algorithms. The UpSet diagram summarizes the intersection of hub genes predicted by all five methods, providing a set of genes that are robustly ranked as hubs regardless of the specific algorithm. For the Delta variant, 12 hub genes were found to be common across all five methods (**Figure 11F**), which includes *CCL5, IGF1, VCL, VWF, CD34, ANGPT1, CD68, HGF, SPP1, CCL2, MMP9*, and *CSF1*. KEGG pathway analysis revealed that these hub genes were primarily involved in Focal adhesion, PI3K-Akt signaling pathway, Rap1 signaling pathway, Ras signaling pathway, MAPK signaling pathway, and TNF signaling pathway (**Figure 11G**). And for Omicron variant infection 7 common hub genes were identified (**Figure 12F**) namely, *VWF, CD34, CD68, IGF1, SPP1, CCL5*, and *MMP9*. KEGG pathway analysis revealed that the hub genes were primarily involved in Focal adhesion, and PI3K-Akt signaling pathway (**Figure 12G**). The AUC values of hub-genes for the Delta and Omicron variant infected cat lung explants is given in **Figures 13A**, **13B**, respectively. A comprehensive list of all enriched KEGG pathways (p ≤ 0.05) associated with hub genes following Delta and Omicron variant infections, as well as KEGG pathways corresponding to significant clusters with an MCODE score > 6 (p ≤ 0.05) for both variants, is provided in **Supplementary Material 3**. **Table 1** represents the details of hub genes after delta and omicron variant infection in cat lung explant culture.

**Table 1:** Details of the hub genes.

Gene symbol and full name	Status	Role	Hub gene	
			Delta (AUC)	Omicron (AUC)
*AGNPT1* (Angiopoietin 1)	↓ (Down)	*ANGPT1* plays a key role in vascular stability and repair by binding to the TEK receptor on endothelial cells, activating pathways that reinforce cell junctions, reduce vessel leakage, and support angiogenesis. It also helps with tissue repair by enabling endothelial cell migration and interaction with the extracellular matrix (losef et al., 2023, Wei et al., 2021).	Yes (0.93)	No
*CCL5* (C-C motif chemokine ligand 5)	↑ (Up)	*CCL5* plays an active role in recruiting a variety of leukocytes including T cells, macrophages, eosinophils, and basophils into inflammatory sites, it also induces the activation and proliferation of natural killer cells to generate CC chemokine-activated killer cells (Soria and Ben-Baruch, 2008).	Yes (0.89)	Yes (0.85)
*CCL2/MCP-1* (C-C motif chemokine ligand 2)	↑ (Up)	*CCL2* (*MCP-1*), a chemokine, plays a critical role in COVID-19 by contributing to inflammation and immune cell recruitment, especially in severe cases (Ansari et al., 2024)	Yes	No
*CD34* (CD34 molecule)	↓ (Down)	Possible adhesion molecule with a role in early hematopoiesis by mediating the attachment of stem cells to the bone marrow extracellular matrix or directly to stromal cells (GeneCards – The Human Gene Database, 2024)	Yes (0.85)	Yes (0.85)
*CD68* (CD68 molecule)	↑ (Up)	*CD68* plays a role in phagocytic activities of tissue macrophages, both in intracellular lysosomal metabolism and extracellular cell-cell and cell-pathogen interactions. (GeneCards – The Human Gene Database, 2024)	Yes (0.89)	Yes (0.93)
*CSF1* (colony stimulating factor 1)	↑ (Up)	*CSF1* regulates the survival, proliferation, and differentiation of mononuclear phagocytes, supporting macrophage-mediated immune defense (Chitu and Stanley, 2006).	Yes (0.78)	No
*HGF* (Hepatocyte Growth Factor)	↓ (Down)	*HGF* gene plays a vital role in countering inflammation and promoting lung tissue repair in severe COVID-19 cases. HGF, a pleiotropic cytokine produced by mesenchymal cells and macrophages, is crucial for preventing lung fibrosis by reducing apoptosis of lung epithelial and endothelial cells. It does so by activating pathways like ERK/MAPK, PI3K/Akt, and STAT3, which support cell survival and tissue regeneration (Perreau et al., 2021)	Yes (0.78)	No
*IGF1* (insulin like growth factor 1)	↓ (Down)	*IGF1* (Insulin-like Growth Factor 1) plays a significant role in immune regulation and inflammation in COVID-19, having both pro-inflammatory and anti-inflammatory effects (Mohamed et al., 2023; Poudel et al., 2020)	Yes (0.74)	Yes (0.78)
*MMP9* (matrix metallopeptidase 9)	↑ (Up)	Matrix metalloproteinase-9 (*MMP9*) plays a multifaceted role in COVID-19, contributing to both lung tissue damage and potential repair mechanisms. Initially, MMP9, produced by neutrophils and monocytes, is upregulated during inflammation, where it breaks down the alveolar-capillary barrier, leading to increased lung permeability and worsening inflammation, but its persistent elevation during recovery phase may aid in tissue remodeling and repair, supporting the reorganization of the extracellular matrix as inflammation subsides (Gelzo et al., 2022)	Yes (0.93)	Yes (0.96)
*SPP1* (secreted phosphoprotein 1)	↑ (Up)	*SPP1* plays a vital role in activating pro-inflammatory monocytes and neutrophils, which are crucial for the immune defense in severe infections. It is essential immunomodulatory factor that recruits immune cells, particularly T cells and macrophages, and promotes antiviral cytokine production, such as IFN-γ (Matacic, 2020; Liukang et al., 2024)	Yes (0.74)	Yes (0.81)
*vWF* (von Willebrand factor)	↓ (Down)	von Willebrand factor (vWF) is a multimeric glycoprotein primarily secreted by endothelial cells, where it plays a central role in initiating platelet adhesion following vascular injury (Rusu and Minshall, 2018). Beyond its well-established function in hemostasis, vWF is a key mediator of thrombosis and thromboinflammation, responding dynamically to changes in blood shear flow (Wagner, 1990). Elevated vWF levels have been associated with poor clinical outcomes in acute lung injury and ARDS (Ware et al., 2004) and are implicated in thrombo-inflammatory processes and coagulopathies observed in SARS-CoV-2 infection (Morici et al., 2020).	Yes (0.85)	Yes (0.78)
*VCL* (Vinculin)	↓ (Down)	Vinculin was identified as a component of focal adhesions and adherens junctions (Bays and DeMali, 2017)	Yes (0.74)	No

### 3.7 Validation by qRT-PCR

**Figure 14:**
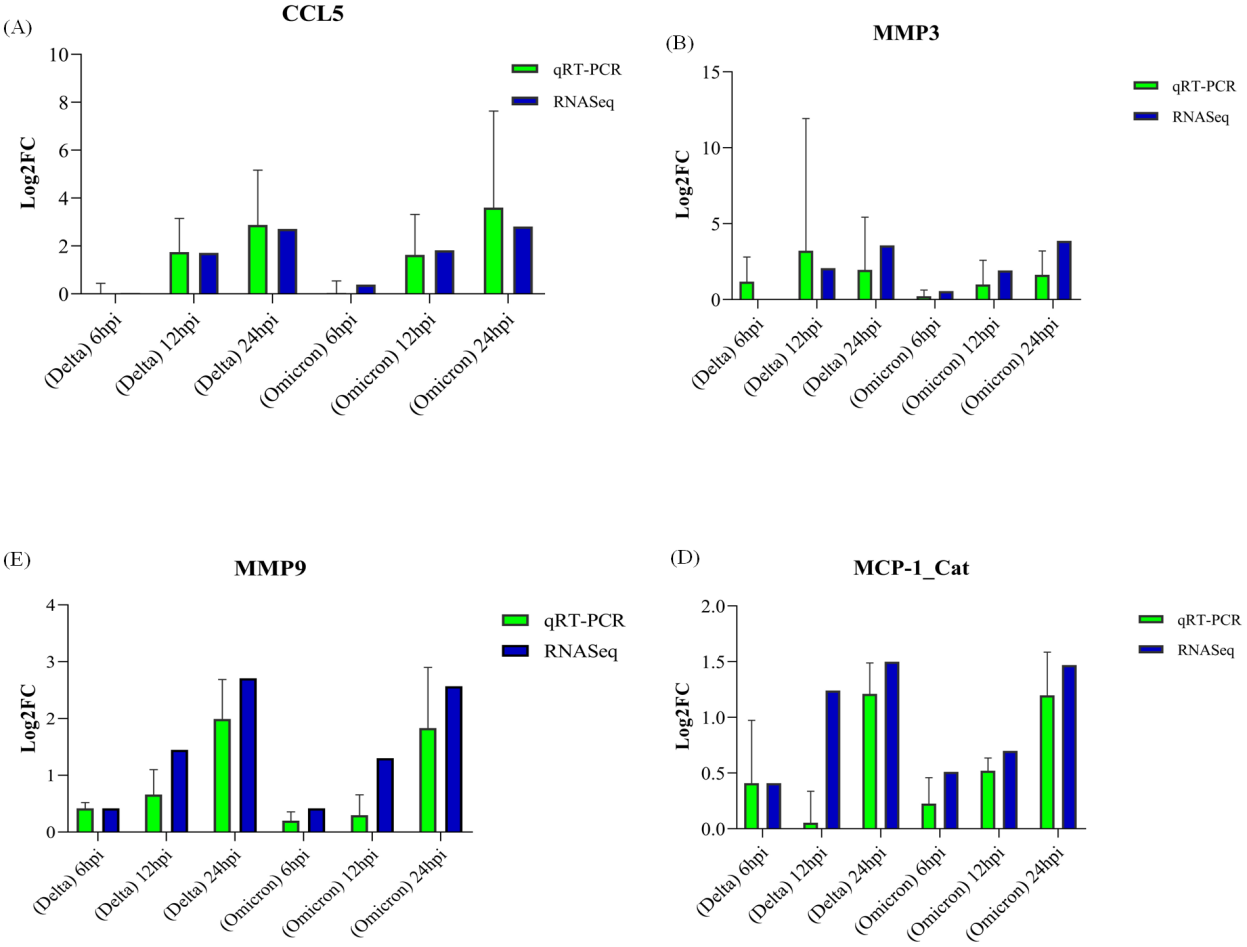
Validation of gene expression by qRT-PCR. Panels **(A–D)** display log2 fold-change (log2FC) values for genes validated in cats: **(A)** CCL5, **(B)** MMP3, **(C)** MMP9, and **(D)** MCP1, with error bars representing standard error (SE).

For validation, four genes were selected among which 3 were identified as hub gene (*MMP9, CCL2/MCP-1, CSF1*) and one was selected randomly (*MMP3*). GAPDH and ACTB were used as housekeeping genes to normalize the qRT-PCR data. Real-time PCR results aligned with the RNA-Seq data. qRT-PCR bar plots with standard error is shown in **Figures 14A–D**.

## 4 Discussion

Domestic cats, living in close proximity to humans, present a potential risk for SARS-CoV-2 transmission and may serve as reservoirs for viral mutations and variant emergence. Cases of reverse zoonosis, where humans transmit the virus to cats, have been well-documented (Barrs et al., 2020; Garigliany et al., 2020; Hosie et al., 2021; Klaus et al., 2021). Experimental studies in cats have demonstrated animal-to-animal transmission of SARS-CoV-2 (Halfmann et al., 2020; Sailleau et al., 2020; Bosco-Lauth et al., 2020; Gaudreault et al., 2020; Shi et al., 2020), underscoring the need to understand the molecular mechanisms of infection in companion animals. Transcriptomic analysis, combined with ex vivo tissue models, offers a powerful approach to studying virus-host interactions by overcoming the limitations of traditional *in vitro* systems and animal models, which struggle to replicate tissue-specific immune responses, particularly for SARS-CoV-2 (Kumar et al., 2020; Gretebeck and Subbarao, 2015). These models preserve tissue architecture and complexity, enabling more accurate insights into immune responses and disease progression (Gordhan et al., 2024). Therefore, in this study we employed an ex vivo lung explant culture model, to investigate the key changes in gene expression in cats infected with the Delta and Omicron variants of SARS-CoV-2 at three distinct time points. COVID-19 induces a profound systemic inflammatory response that significantly disrupts the extracellular matrix (ECM)—a critical structure maintaining tissue homeostasis and facilitating repair (Zhou et al., 2018; Sarker et al., 2021). In the respiratory system, the ECM provides structural integrity to the upper airways and forms a specialized basement membrane (BM) in the lower airways and vasculature, essential for normal tissue maintenance and organ development (Rozario and DeSimone, 2010). Consistent with this, our results revealed significant downregulation of surfactant-related genes, essential for lung function and homeostasis, likely contributing to respiratory distress (Whitsett et al., 2010). Surfactant proteins such as *SFTPB, SFTPC, SFTPD*, and *SFTA2* play critical roles in reducing alveolar surface tension and stabilizing alveolar structures (Perez-Gil and Weaver, 2010; Nogee et al., 2000). Their suppression could exacerbate alveolar collapse and impair gas exchange. Simultaneously, genes involved in cell-matrix adhesion and extracellular matrix (ECM) integrity were diminished, including laminins (*LAMA2*), elastin (*ELN*), and various collagens (*COL16A1, COL13A1, COL8A1*). Disruption of these ECM components compromises lung structure, leading to epithelial dysfunction, tissue damage, and mechanical instability (Leng et al., 2020). Downregulation of *FBLN5*, critical for elastogenesis and preservation of lung tissue elasticity (Papke and Yanagisawa, 2014), was also observed, further indicating compromised lung function. Notably, elastin (*ELN*) downregulation, particularly more pronounced in Delta infection, reflects loss of lung elasticity, consistent with pathological processes observed in emphysema (Vlahovic et al., 1999; Deslee et al., 2009). Suppression of tight junction genes such as *CLDN11, CLDN16*, and *CLDN18* suggests impaired epithelial barrier integrity, potentially enhancing vascular permeability and pulmonary edema, as reported in COVID-19-related lung injury (Hashimoto et al., 2022). In addition, downregulation of adhesion molecules such as *POSTN*, *CAV1*, and *TSPAN7* may contribute to weakened ECM attachment. *POSTN*, for example, is critical for collagen fibrillogenesis and cross-linking in connective tissues (Canty and Kadler, 2005; Maruhashi et al., 2010). Pathway analysis highlighted disruptions in ECM organization, cell adhesion, and angiogenesis (Huang et al., 2023), indicating a broad impact on lung structure and repair mechanisms. Furthermore, ADAM and ADAMTS proteases—key regulators of ECM remodeling—were downregulated (*ADAMTS2, ADAMTS5, ADAMTS8, ADAMTS9, ADAM33*), suggesting impaired collagen processing and tissue regeneration (Wang et al., 2023). Both Delta and Omicron variants triggered activation of common inflammatory pathways, such as cytokine-cytokine receptor interaction and chemokine signaling (Blanco-Melo et al., 2020), both of which are known drivers of severe COVID-19 complications (Valle et al., 2020). However, Delta induced broader and more severe changes, including variant-specific activation of lung development and alveolus formation pathways, suggesting greater structural damage and compromised repair (Saito et al., 2022; Rajaiah et al., 2024). A significant downregulation of growth and secreted factors such as *ANGPT1, HGF, IGF1*, and *WNT7A* was also observed. *ANGPT1* reinforces endothelial junctions and promotes vascular stability (Iosef et al., 2023; Wei et al., 2021). Its suppression may weaken vascular integrity and enhance leakage. *HGF* acts as a critical tissue-repair factor (Perreau et al., 2021), and *IGF1* regulates immune responses and cellular repair, with reduced levels associated with worsened outcomes in respiratory diseases including ARDS (Ahasic et al., 2012; Mohamed et al., 2023; Poudel et al., 2020).

In the context of inflammation and vascular injury, elevated *ANGPT2* levels observed in our study are notable. *ANGPT2* has been previously linked with vascular dysfunction and poor outcomes in COVID-19 and H1N1 infections (Parikh et al., 2006). Similarly, upregulation of SPP1, a mediator of monocyte and neutrophil activation (Liukang et al., 2024), and increased chemokines like *CCL5* and *MCP-1* (Pérez-García et al., 2022; Ansari et al., 2024) suggest enhanced immune cell recruitment to lung tissues, potentially contributing to hyperinflammation. Tissue remodeling signatures were evident through upregulation of genes such as *MMP9, MMP12, COLGALT1, FHOD3*, and *WNT5A*. Notably, MMP9, which can degrade ECM components and promote alveolar capillary destruction, was upregulated in both variants (Salomão et al., 2023). Upregulation of *MMP12*, previously associated with airway remodeling and fibrosis in asthma (Pouniotis et al., 2006; Meidaninikjeh et al., 2021; Abd-Elaziz et al., 2021), also points to potential long-term lung damage. *COLGALT1*, important for collagen triple helix formation (Tvaroška, 2024), was consistently elevated, further supporting extensive ECM alterations. Pathway analysis highlighted disruptions in ECM organization, cell adhesion, and angiogenesis, further emphasizing the virus’s impact on tissue structure and repair mechanisms (Huang et al., 2023). Both Delta and Omicron variants demonstrated activation of common inflammatory pathways, such as cytokine-cytokine receptor interaction and chemokine signaling, which play critical roles in immune responses to viral infections (Blanco-Melo et al., 2020). These pathways have been implicated in hyperinflammatory states driving severe complications in COVID-19 (Valle et al., 2020). However, the Delta variant induced broader and more severe changes, including variant-specific activation of lung development and alveolus formation pathways, suggesting greater structural damage and compromised tissue repair (Saito et al., 2022).

Further insights into the host response were gained through hub gene identification and Area Under the Curve (AUC) analysis. Key hub genes, such as *CCL5*, *MMP9*, and *SPP1*, were upregulated, reflecting heightened immune responses and inflammation. *CCL5* enhances immune cell recruitment and antiviral defenses (Pérez-García et al., 2022), while *MMP9* contributes to both lung tissue damage and repair during inflammation (Gelzo et al., 2022). Conversely, downregulated hub genes like *ANGPT1* and *HGF* suggest weakened vascular stability and impaired tissue repair (Iosef et al., 2023; Wei et al., 2021; Perreau et al., 2021). Interestingly, *VWF* was downregulated in cats, contrasting with its elevation in severe human COVID-19 cases, potentially indicating a reduced risk of vascular injury (Wang et al., 2021; Wibowo et al., 2022). The detailed roles of these hub genes are summarized in **Table 1**.

The pronounced disruption of ECM-related genes and pathways underscores the structural vulnerabilities caused by SARS-CoV-2, likely contributing to respiratory distress and compromised lung function. The Delta variant’s more aggressive response, as reflected in broader gene dysregulation, may explain its increased pathogenicity. While recovery-associated gene expression was not prominent within the 24-hour post-infection window, seropositivity studies suggest that cats may develop immune responses over time (Bosco-Lauth et al., 2020). This raises the possibility that reparative pathways could emerge at later stages of infection, warranting further investigation. Overall, our findings demonstrate the utility of the ex vivo lung explant culture model in studying zoonotic viruses and their molecular impact. The identification of key hub genes involved in ECM integrity, immune modulation, and inflammation provides significant insights into the pathogenesis of SARS-CoV-2. Therapeutic strategies aimed at restoring surfactant production, preserving ECM integrity, and modulating angiogenesis hold promise for mitigating lung injury. These findings contribute to our understanding of viral pathogenesis and offer a foundation for developing targeted interventions to improve outcomes in both veterinary and human medicine. Although we observed a consistent decline in Ct values from 6 to 24 hours post-infection, indicating progressive viral RNA accumulation, the overall change was modest, suggesting limited replication in our ex vivo feline lung explant model. For instance, Hui et al. (2020) reported a ~2-log_10_ increase in viral titres in human bronchial explants over 72 hours, whereas Caco-2 cells showed increases exceeding 4-log_10_. Similarly, Chu et al. (2020) documented a 1.20 to 2.04-log increase in viral titres in human lung explants between 2 and 48 hours post-infection. Furthermore, our 24-hour observation window may have been insufficient to detect later stages of viral amplification. Future studies incorporating extended timepoints and assessing additional respiratory tissues, such as trachea and nasal mucosa, could provide a more comprehensive understanding of tissue-specific viral dynamics and host responses in cats.

## 5 Conclusion

In conclusion, our RNA-seq analysis highlights transcriptional responses in cat lung explant cultures following SARS-CoV-2 infection. The observed downregulation of key genes related to extracellular matrix (ECM) organization, surfactant homeostasis, and cell adhesion suggests that the virus may affect molecular components critical for lung structure and function. These molecular alterations provide valuable insights into the host’s transcriptomic response to SARS-CoV-2, with the Delta variant causing more pronounced changes compared to the Omicron variant. These findings offer valuable insight into the feline host response to SARS-CoV-2 infection. However, they are specific to cat lung tissue and due to species-specific differences in ACE2 receptor structure and TMPRSS2 expression, direct extrapolation of these results to other animal models or humans should be approached with caution. Moreover, as the study is based solely on transcriptomic data without histopathological validation, therefore the observed molecular alterations should be considered indicative rather than demonstrative of tissue-level damage or functional impairment. Future studies incorporating histopathological examination and viral titration will be essential to confirm the biological consequences of these transcriptomic changes and to better characterize virus-induced effects on lung tissue. Despite these limitations, the identification of dysregulated hub genes provides a foundation for the development of targeted therapeutic strategies and prognostic biomarkers. For instance, interventions aimed at restoring surfactant production, preserving ECM composition, and modulating angiogenic signaling may hold potential in mitigating virus-associated molecular effects. Finally, our findings highlight the relevance of the ex vivo cat lung explant culture model as a powerful and biologically meaningful platform for studying SARS-CoV-2 infection dynamics and for advancing research on emerging zoonotic viruses.

## Data Availability

The original contributions presented in the study are publicly available. This data can be found here: NCBI/PRJNA1328388.
