# Supplementary material for: Comparative temporal transcriptome analyses of SARS-CoV-2 delta and omicron variants ex-vivo infection in cat lung explant culture
Source: Front Cell Infect Microbiol. 2025 Oct 22;15:1553464. doi: 10.3389/fcimb.2025.1553464 (PMC12586921; doi:10.3389/fcimb.2025.1553464)

Supplementary material 1

Table 1: Ct values obtained for Delta and Omicron variant infected cat lung explants culture at different time points (control, 0hpi, 6hpi, 12hpi, and 24hpi) post infection

Variant of SARS-CoV-2	Time point post infection	FAM (Target E gene)	Texas Red (Target RdRp gene)	Cy5 (Target N gene)
Delta Variant	Control_rep1	NA	NA	NA
	Control_rep2	NA	NA	NA
	Control_rep3	NA	NA	NA
	0hpi_rep1	22.58	21.35	21.17
	0hpi_rep2	23.45	22.15	22.08
	0hpi_rep3	23.28	21.76	21.72
	6hpi_rep1	19.49	19.19	17.42
	6hpi_rep2	19.38	19.55	17.57
	6hpi_rep3	19.19	19.02	16.45
	12hpi_rep1	19.13	18.56	16.62
	12hpi_rep2	19.33	19.57	17.09
	12hpi_rep3	19.40	19.30	17.18
	24hpi_rep1	18.82	18.16	16.14
	24hpi_rep2	18.18	18.26	15.68
	24hpi_rep3	19.27	19.40	16.94
Omicron Variant	Control_rep1	NA	NA	NA
	Control_rep2	NA	NA	NA
	Control_rep3	NA	NA	NA
	0hpi_rep1	24.51	23.22	22.31
	0hpi_rep2	24.38	22.86	21.84
	0hpi_rep3	25.41	23.59	22.57
	6hpi_rep1	22.02	20.46	17.37
	6hpi_rep2	22.83	21.50	18.39
	6hpi_rep3	21.13	19.62	16.59
	12hpi_rep1	22.38	20.40	16.66
	12hpi_rep2	22.09	21.11	18.27
	12hpi_rep3	21.15	20.55	17.29
	24hpi_rep1	21.24	20.25	15.88
	24hpi_rep2	21.48	20.36	16.48
	24hpi_rep3	21.11	20.15	16.51

Table 2: Transcriptomics raw data statistics

Species	SARS-CoV-2 Variant	Hours post infection (hpi)	Replicate No	Reads in millions	Raw data in GB
CAT	DELTA	Control	1	7.22	11
			2	6.3	10
			3	8.5	14
		6 hpi	1	5.7	9
			2	6.9	11
			3	7.2	11
		12 hpi	1	7.7	12
			2	8.0	13
			3	7.9	13
		24hpi	1	8.2	13
			2	7.0	11
			3	6.3	10
	OMICRON	6hpi	1	6.6	10
			2	6.3	10
			3	6.6	11
		12hpi	1	8.9	14
			2	6.5	10
			3	6.9	11
		24hpi	1	6.7	11
			2	7.5	12
			3	9.1	15

Table 3: List of primers used for qRT-PCR

Primer Name	Sequence (5'-3')	Amplicon size	AT	References
MMP9-Forward	GCCCCTACAGTGTCTTTGGA	102bp	59°C	Tamamoto *et al.*, 2017
MMP9-Reverse	TCCCATCCTTGAAGAAATGC			
MCP1-Forward	TGCAGAGGCTGGTGAGCTATAA	102bp	59°C	Zini *et al.*, 2010
MCP1-Reverse	TTGGGTCAGCGCAGATCTC			
MMP3-Forward	TGACTCGAAGGTTGATGCTG	99bp	59°C	Fujihara *et al.*, 2018
MMP3-Reverse	TGTCACTTTCTTTGCGTTGG			
CCL5-Forward	CTACACCAGCAGCAGTGTTCC	67bp	59°C	Designed

CCL5-Reverse	ACACACCTGGCGCTTCCTC			
GAPDH-Forward	AAATTCCACGGCACAGTCAAG	60bp	59°C	Cook *et al.*, 2022
GAPDH-Reverse	TGATGGGCTTTCCATTGATGA			
ACTB-Forward	TGCGTGACATCAAGGAGAAG	175bp	59°C	Scimeca *et al.*, 2023
ACTB-Reverse	AGGAAGGAAGGCTGGAAGAG			

Table 4: Percentage of reads successfully aligned to the reference (*Felis catus*)

Sample Code	Meaning	Total Reads	Mapped Reads	Alignment Percentage
**C1CC6T**	Control_rep1	70008104	66,153,799	94.49448738
**C1Delta6T**	Delta 6hpi_rep1	51739624	45,859,481	88.63512615
**C1Delta12T**	Delta 12hpi_rep1	75357514	70,914,716	94.10437292
**C1Delta24T**	Delta 24hpi_rep1	80264226	76,598,088	95.43241344
**C1Om6T**	Omicron 6hpi_rep1	64567236	61,537,062	95.30694794
**C1Om12T**	Omicron 12hpi_rep1	84213462	79,474,090	94.37219194
**C1Om24T**	Omicron 24hpi_rep1	63066180	59,207,426	93.88142107
**C2CC6T**	Control_rep2	59275322	55,753,872	94.05916344
**C2Delta6T**	Delta 6hpi_rep2	64416018	59,448,495	92.28837306
**C2Delta12T**	Delta 12hpi_rep2	74484924	63,275,061	84.9501585
**C2Delta24T**	Delta 24hpi_rep2	65804816	61,898,998	94.06454081
**C2Om6T**	Omicron 6hpi_rep2	57904328	52,883,896	91.32978108
**C2Om12T**	Omicron 12hpi_rep2	57217206	51,413,482	89.85668052
**C2Om24T**	Omicron 24hpi_rep2	72283832	68,792,479	95.16993925
**C3CC6T**	Control_rep3	82193474	78,161,992	95.09513128
**C3Delta6T**	Delta 6hpi_rep3	69695528	66,391,341	95.25911189
**C3Delta12T**	Delta 12hpi_rep3	76714366	72,928,271	95.0646858
**C3Delta24T**	Delta 24hpi_rep3	61278456	57,935,936	94.54535865
**C3Om6T**	Omicron 6hpi_rep3	63560598	60,271,887	94.82586523
**C3Om12T**	Omicron 12hpi_rep3	65791954	62,298,150	94.68961813
**C3Om24T**	Omicron 24hpi_rep3	88689890	84,282,444	95.03049784

Table 5: Sequence stats for RNA-Seq sample

Samples	Meaning	Reads	Bases	Q20(%)	Q30(%)
**C1CC6T**	Control_rep1	72220062	11482989858	96.58	91.97
**C1Delta6T**	Delta 6hpi_rep1	56965672	9057541848	90.41	83.83
**C1Delta12T**	Delta 12hpi_rep1	77420856	12309916104	96.66	92.01
**C1Delta24T**	Delta 24hpi_rep1	82248532	13077516588	97.28	93
**C1Om6T**	Omicron 6hpi_rep1	66028232	10498488888	96.53	91.95
**C1Om12T**	Omicron 12hpi_rep1	89304810	14199464790	96.32	91.76
**C1Om24T**	Omicron 24hpi_rep1	67014286	10655271474	96.41	91.88
**C2CC6T**	Control_rep2	62995096	10016220264	96	91.35
**C2Delta6T**	Delta 6hpi_rep2	69873952	11109958368	95.78	90.87
**C2Delta12T**	Delta 12hpi_rep2	80667626	12826152534	95.57	90.11
**C2Delta24T**	Delta 24hpi_rep2	70144190	11152926210	96.53	92.05
**C2Om6T**	Omicron 6hpi_rep2	63137062	10038792858	95.89	91.02
**C2Om12T**	Omicron 12hpi_rep2	65454872	10407324648	95.31	90.23
**C2Om24T**	Omicron 24hpi_rep2	75549122	12012310398	97.32	93.13
**C3CC6T**	Control_rep3	85082416	13528104144	96.82	92.37
**C3Delta6T**	Delta 6hpi_rep3	72172714	11475461526	97.02	92.73
**C3Delta12T**	Delta 12hpi_rep3	79365318	12619085562	96.67	92.27
**C3Delta24T**	Delta 24hpi_rep3	63524094	10100330946	96.6	91.96
**C3Om6T**	Omicron 6hpi_rep3	66343294	10548583746	96.86	92.5
**C3Om12T**	Omicron 12hpi_rep3	68423352	10879312968	96.47	91.95
**C3Om24T**	Omicron 24hpi_rep3	91620078	14567592402	97.05	92.68

PRIMARY QC

Isolated RNA was quantified using Nanodrop 2000 (Thermofisher Scientific, Massachusetts, USA). The integrity of RNA was evaluated on 1% agarose (Lonza, Belgium) gel. RNA work and DNA work are always done separately. RNA gel apparatus is only for RNA work, the loading dye, as well as the markers, are for RNA work only, and the nuclease-free TAE buffer is purchased for RNA work and is diluted only in DEPC water.

The obtained results are presented below;

1) RNA QC by Nanodrop

Table 1: RNA quantification in ng/µl using Nanodrop ratios

Sr. No	Sample ID	Meaning	Nanodrop Conc. (ng/ul)	260/280	260/230
1.	C1CC6T	Control_rep1	104.1	1.88	1.99
2.	C1δ6T	Delta 6hpi_rep1	53.4	1.82	1.62
3.	C1δ12T	Delta 12hpi_rep1	45.8	1.61	1.1
4.	C1δ24T	Delta 24hpi_rep1	46.4	1.55	1.07
5.	C1Om6T	Omicron 6hpi_rep1	46.7	1.71	1.7
6.	C1Om12T	Omicron 12hpi_rep1	62.2	1.69	0.69
7.	C1Om24T	Omicron 24hpi_rep1	44.8	1.7	1.53
8.	C2CC6T	Control_rep2	58.1	1.85	0.18
9.	C2δ6T	Delta 6hpi_rep2	70.6	1.77	1.59
10.	C2δ12T	Delta 12hpi_rep2	66.3	1.99	1.96
11.	C2δ24T	Delta 24hpi_rep2	33.4	1.56	1.57
12.	C2Om6T	Omicron 6hpi_rep2	57.8	1.78	1.75
13.	C2Om12T	Omicron 12hpi_rep2	82.2	1.78	1.6
14.	C2Om24T	Omicron 24hpi_rep2	48.4	1.68	1.3
15.	C3CC6T	Control_rep3	56.6	1.79	1.91
16.	C3δ6T	Delta 6hpi_rep3	52.9	1.76	1.85
17.	C3δ12T	Delta 12hpi_rep3	53	1.8	1.33
18.	C3δ24T	Delta 24hpi_rep3	51.6	1.83	1.41
19.	C3Om6T	Omicron 6hpi_rep3	55.4	1.73	0.59
20.	C3Om12T	Omicron 12hpi_rep3	52.5	1.77	0.98
21.	C3Om24T	Omicron 24hpi_rep3	70.1	1.83	1.48

2) RNA QC BY AGAROSE GEL ELECTROPHORESIS

Electrophoresis Condition

Total RNA---Gel Conc.1% : 80v Run Time : 30 min

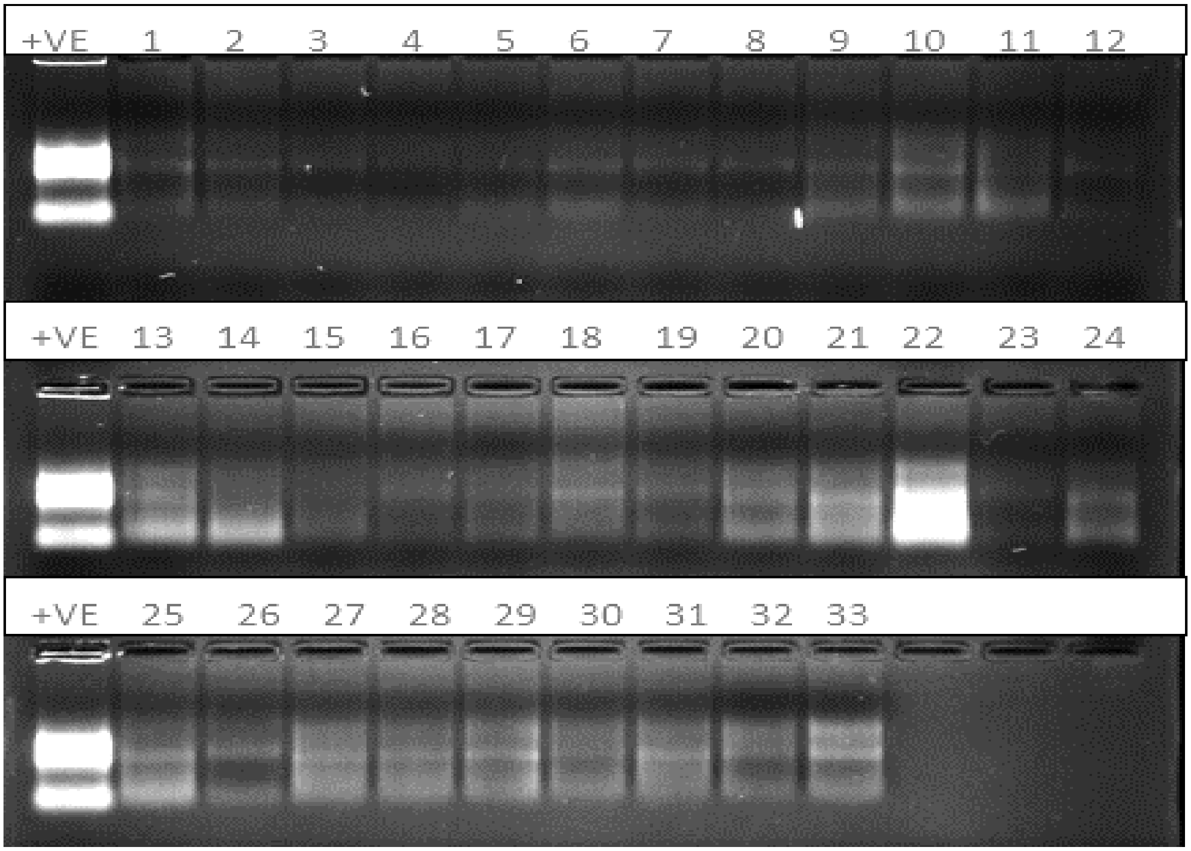

Fig.1. Agarose Gel Electrophoresis Image (1%)

3) LIBRARY QC PROFILE

1) C1CC6T

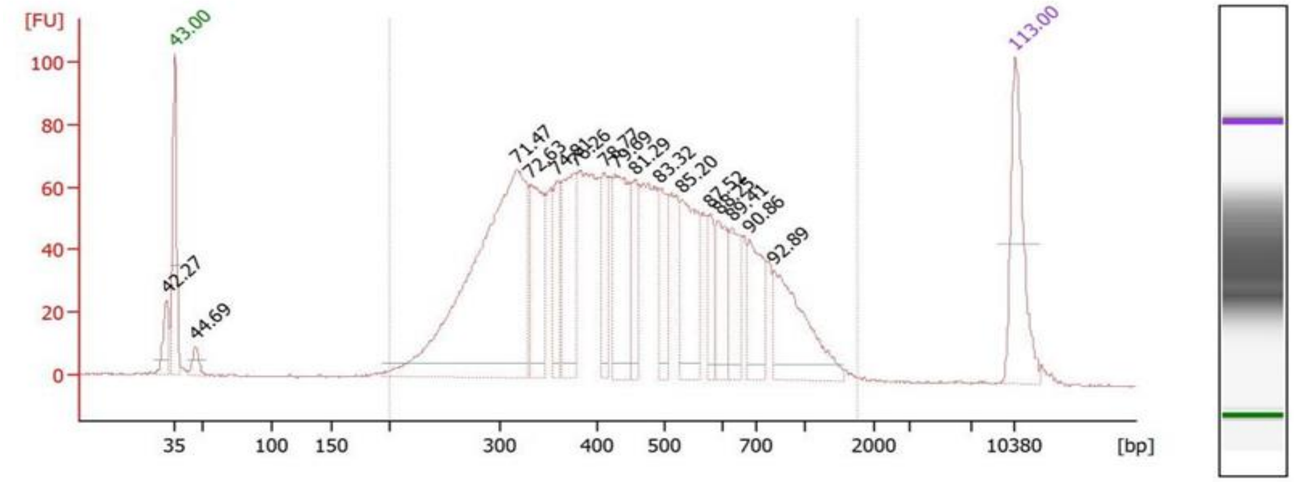

From [bp]	To [bp]	Corr. Area	% of Total	Average Size [bp]	Size distribution in CV [%]	Conc. [pg/µl]	Molarity [pmol/l]	Co lor
200	1,748	2,162.4	97	479	42.2	2,019.47	7,679.5	

2) C1δ6T

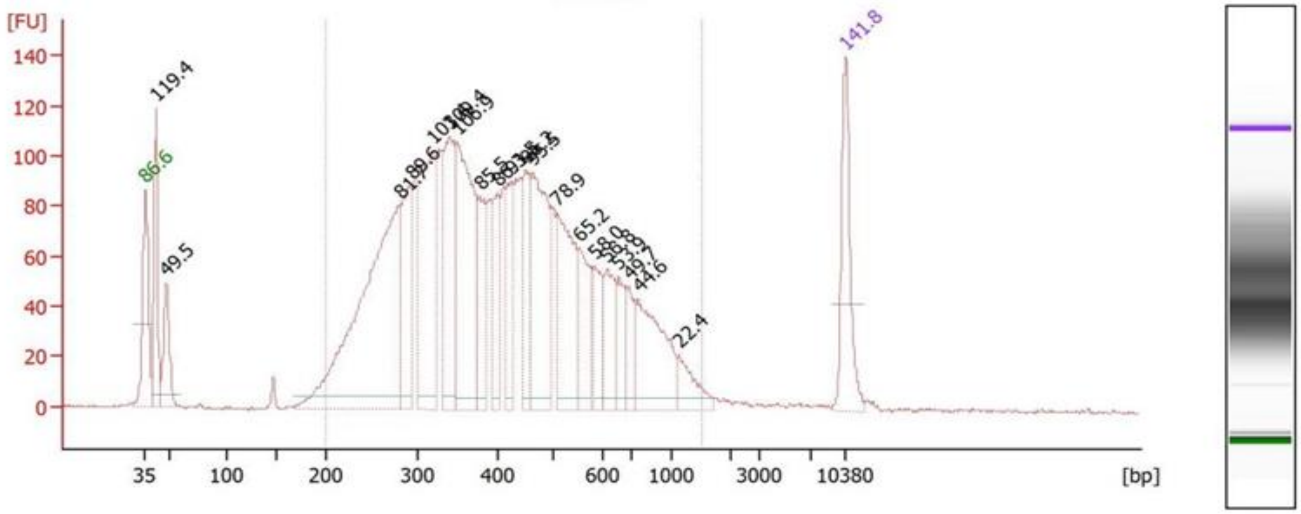

From [bp]	To [bp]	Corr. Area	% of Total	Average Size [bp]	Size distribution in CV [%]	Conc. [pg/µl]	Molarity [pmol/l]	Co lor
200	1,538	3,226.7	92	444	43.7	2,720.66	11,255.8	

3) C1δ12T

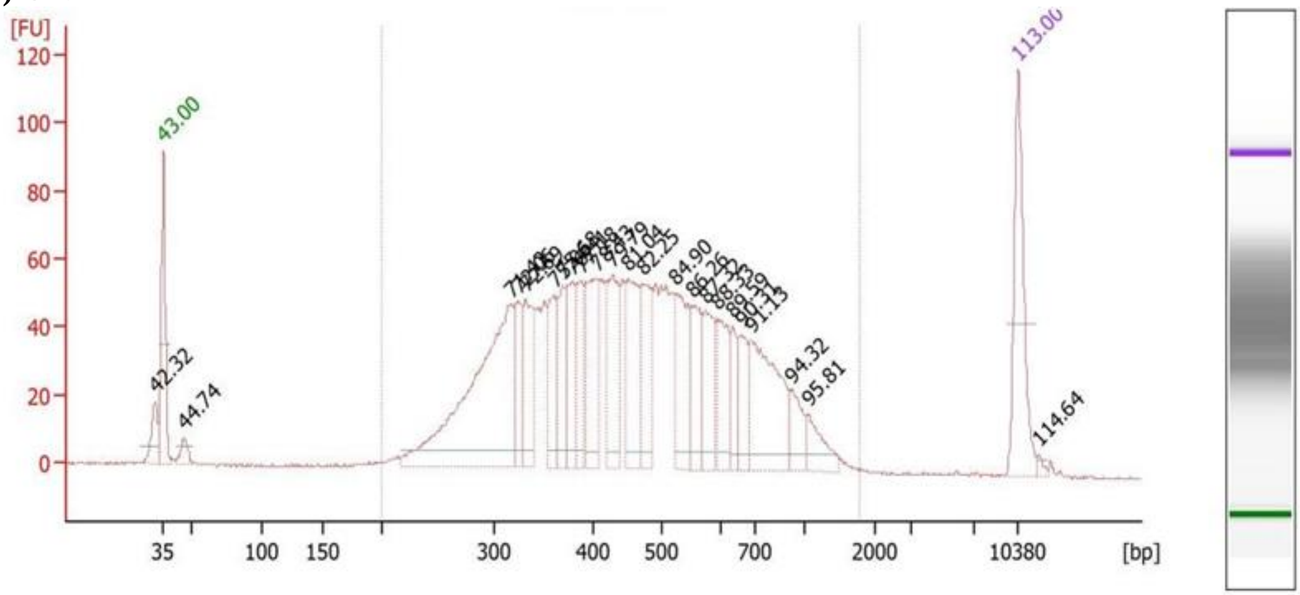

From [bp]	To [bp]	Corr. Area	% of Total	Average Size [bp]	Size distribution in CV [%]	Conc. [pg/µl]	Molarity [pmol/l]	Color
200	1,788	1,751.7	96	491	41.3	1,898.25	6,982.9	

4) C1δ24T

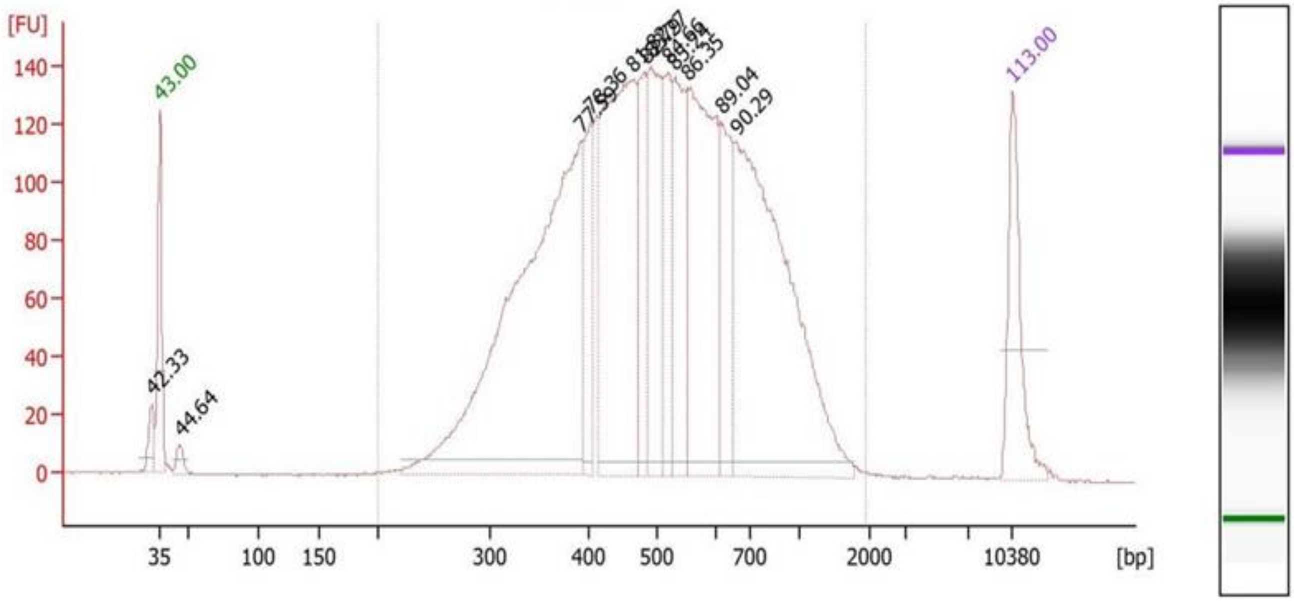

From [bp]	To [bp]	Corr. Area	% of Total	Average Size [bp]	Size distribution in CV [%]	Conc. [pg/µl]	Molarity [pmol/l]	Co lor
200	1,932	3,717.7	98	544	40.6	2,746.58	9,049.7	

5) C1Om6T

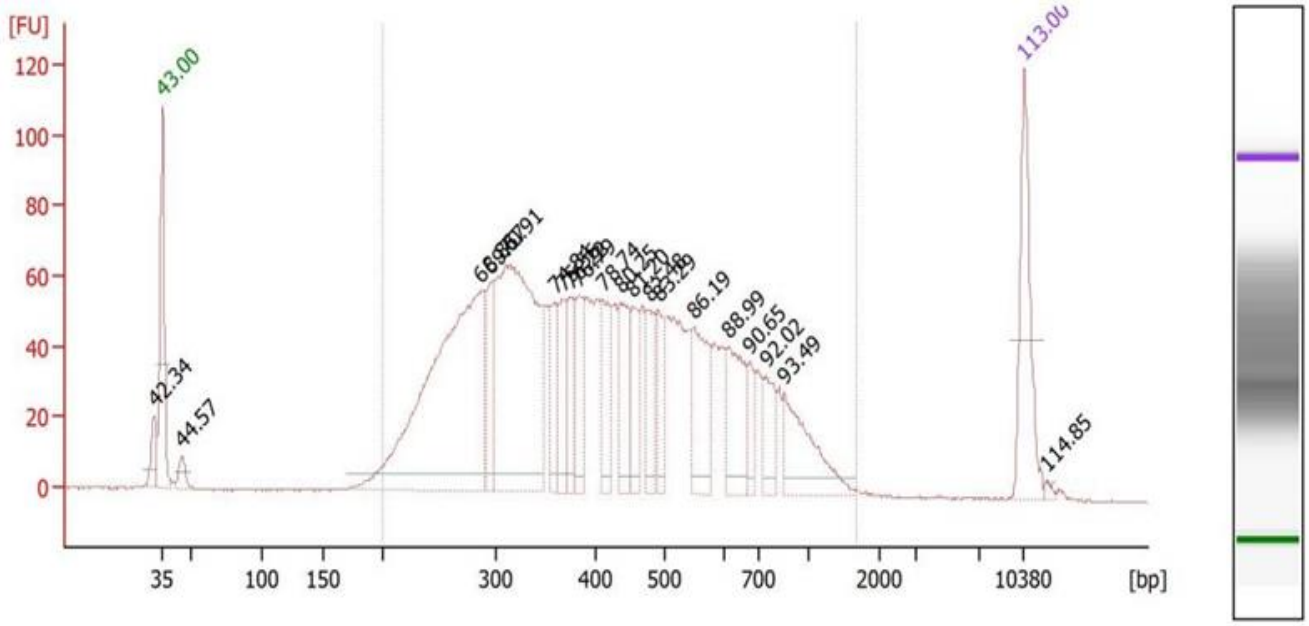

From [bp]	To [bp]	Corr. Area	% of Total	Average Size [bp]	Size distribution in CV [%]	Conc. [pg/µl]	Molarity [pmol/l]	Co lor
200	1,682	2,133.1	96	452	44.7	2,225.71	9,186.1	

6) C1Om12T

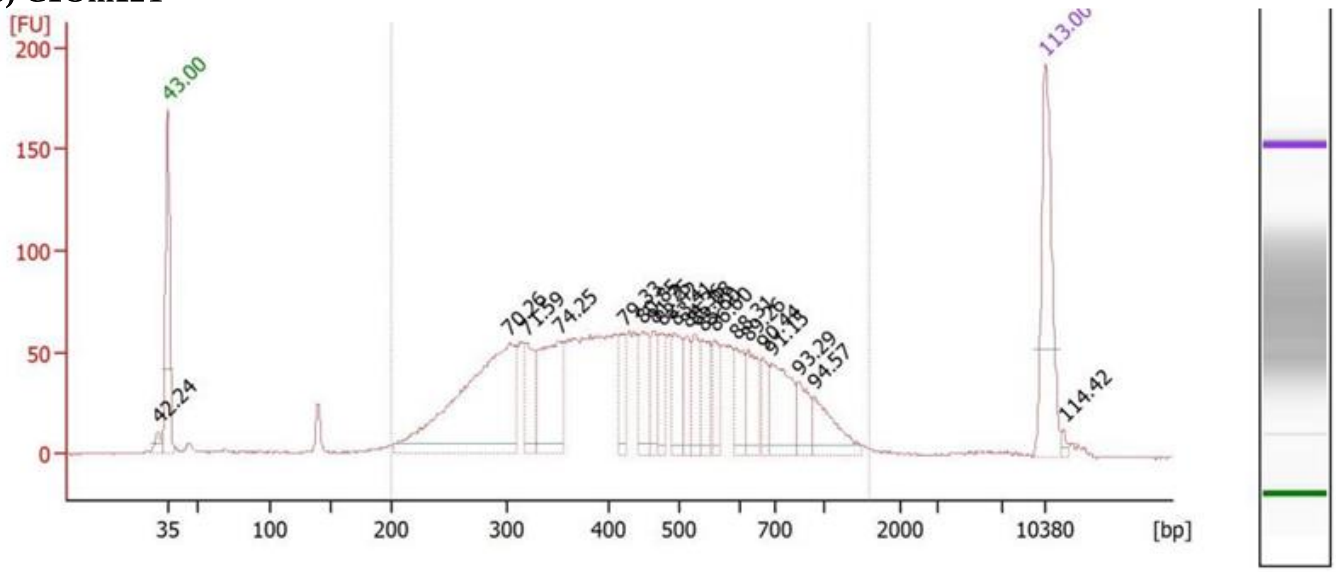

From [bp]	To [bp]	Corr. Area	% of Total	Average Size [bp]	Size distribution in CV [%]	Conc. [pg/µl]	Molarity [pmol/l]	Color
200	1,582	2,181.4	92	481	42.8	1,401.26	5,406.1	

7) C1Om24T

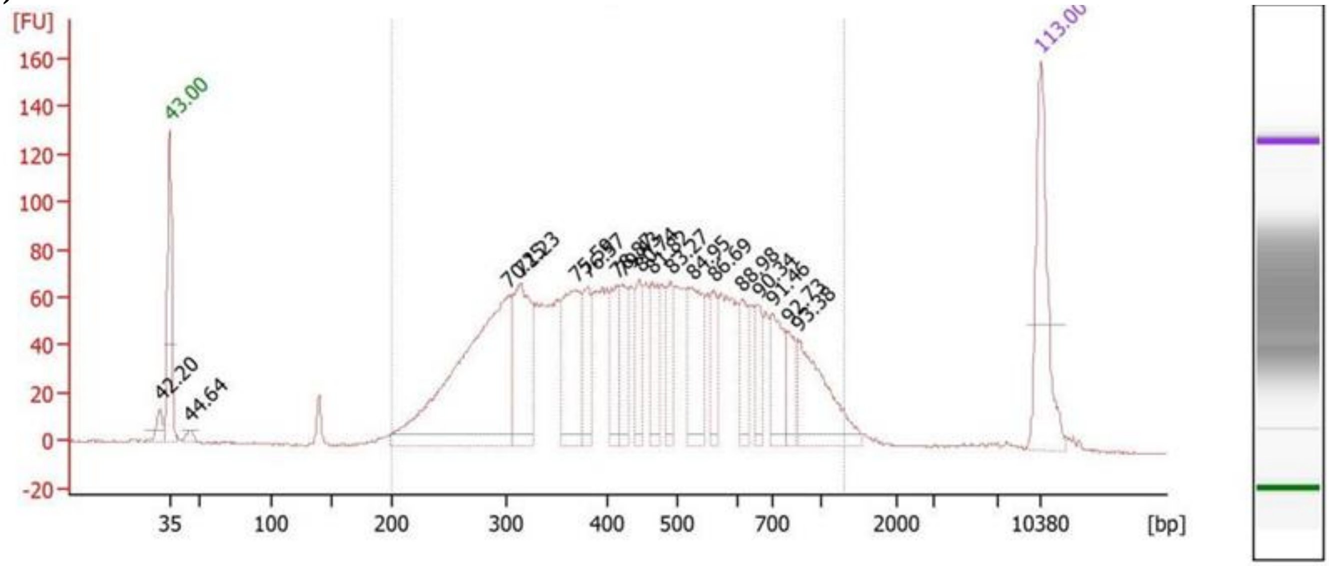

From [bp]	To [bp]	Corr. Area	% of Total	Average Size [bp]	Size distribution in CV [%]	Conc. [pg/µl]	Molarity [pmol/l]	Color
200	1,311	2,483.2	95	481	40.6	1,674.39	6,425.1	

8) C2CC6T

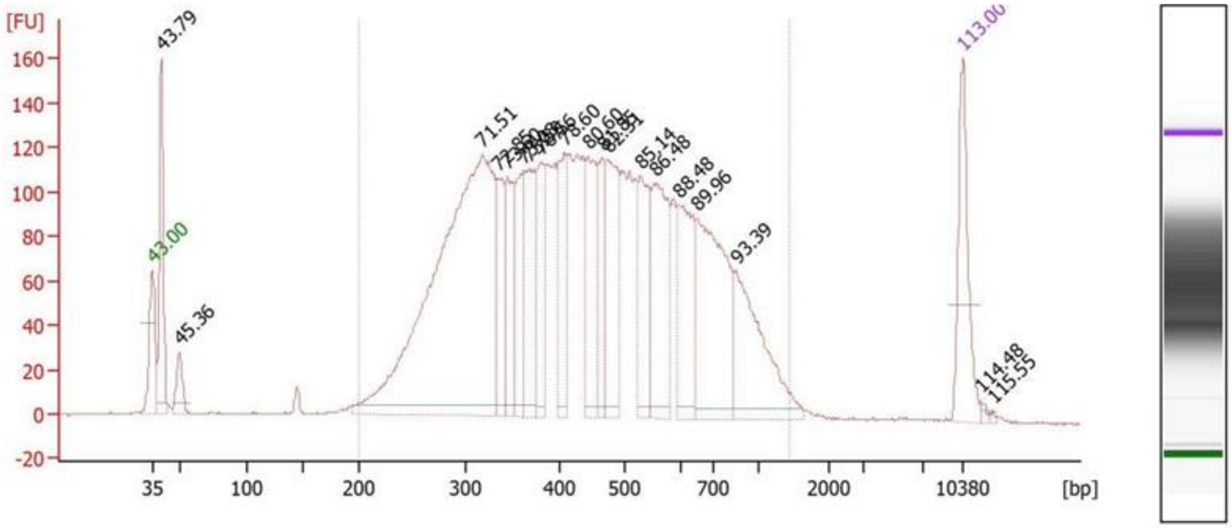

From [bp]	To [bp]	Corr. Area	% of Total	Average Size [bp]	Size distribution in CV [%]	Conc. [pg/µl]	Molarity [pmol/l]	Co lor
200	1,444	4,085.4	93	477	40.4	2,973.80	11,362.0	

9) C2δ6T

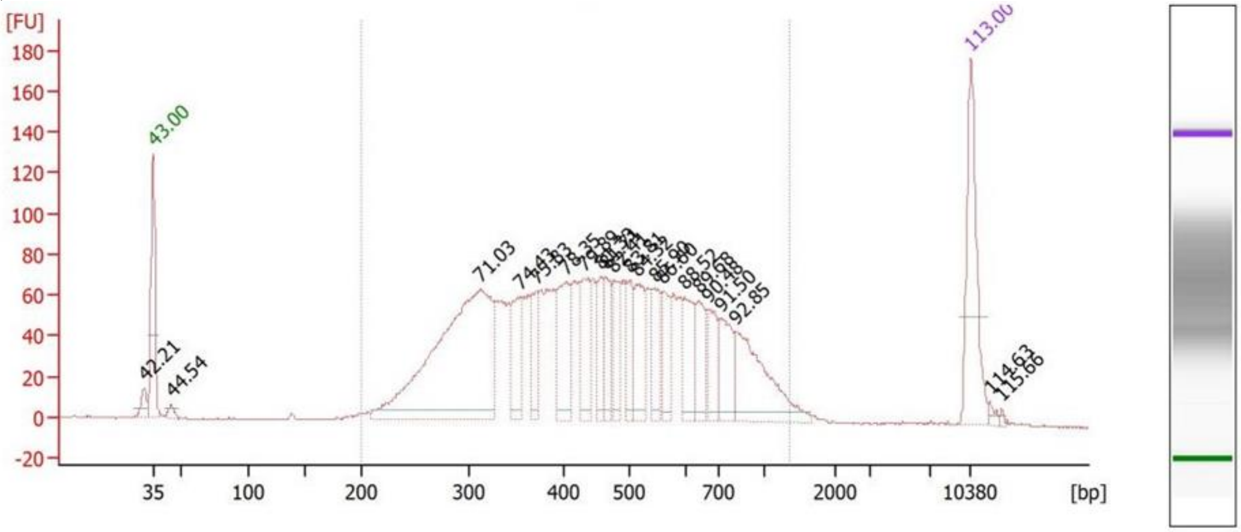

... **Region table for sample 4 :** C2delta6T

From [bp]	To [bp]	Corr. Area	% of Total	Average Size [bp]	Size distribution in CV [%]	Conc. [pg/µl]	Molarity [pmol/l]	Co lor
200	1,357	2,378.6	96	484	39.7	1,602.99	6,042.2	

10) C2δ12T

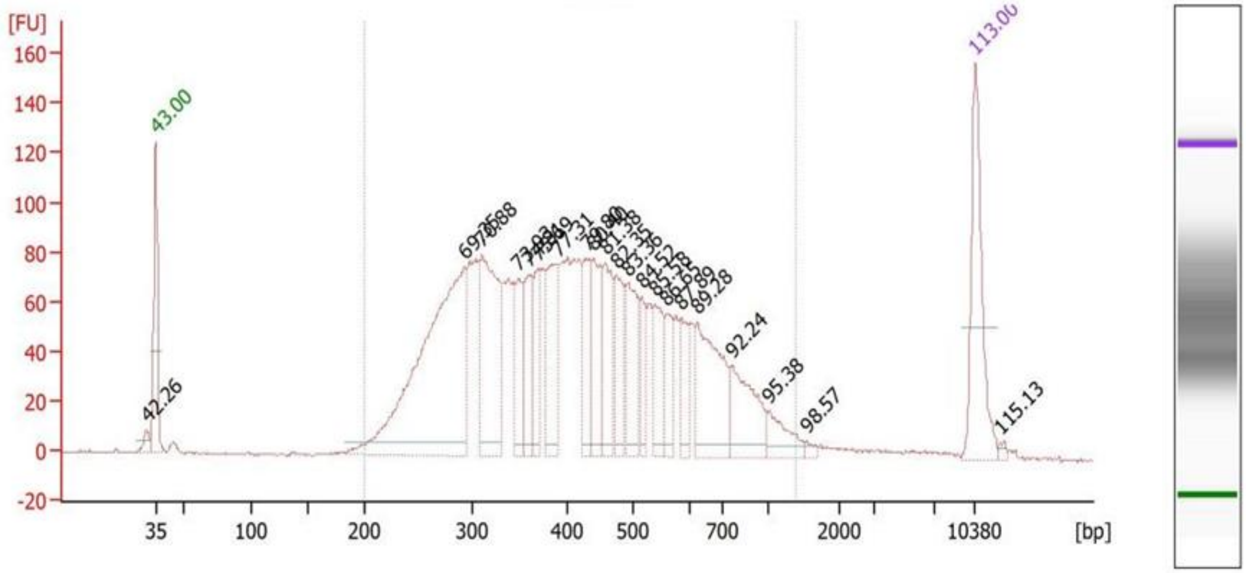

From [bp]	To [bp]	Corr. Area	% of Total	Average Size [bp]	Size distribution in CV [%]	Conc. [pg/µl]	Molarity [pmol/l]	Co lor
200	1,383	2,632.9	97	447	39.6	1,897.90	7,661.1	

11) C2δ24T

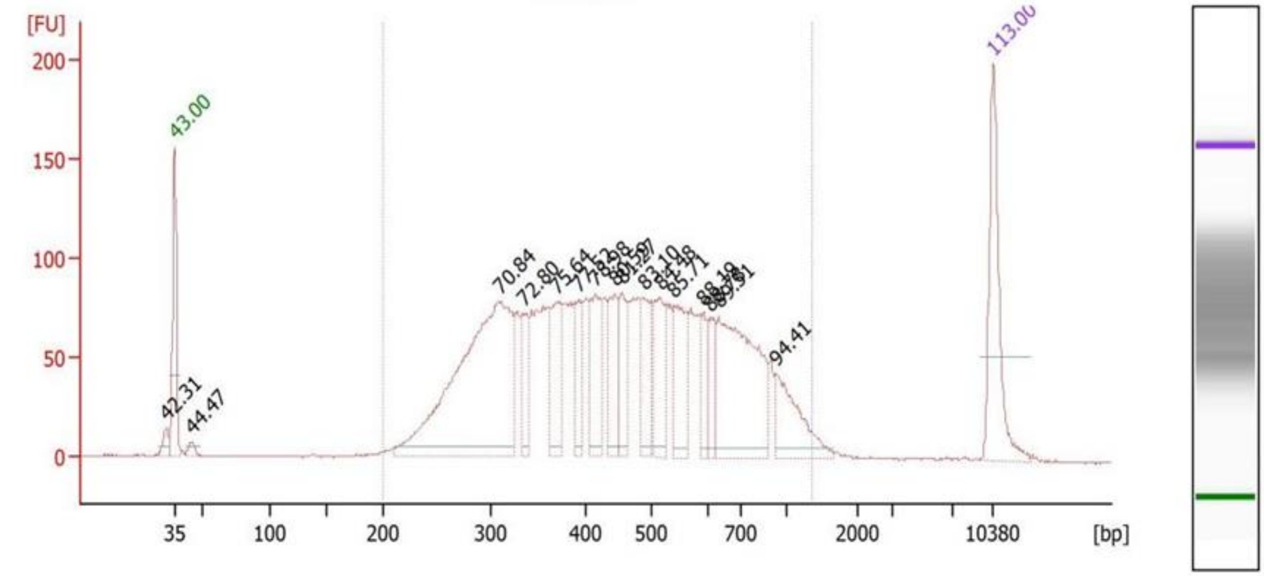

From [bp]	To [bp]	Corr. Area	% of Total	Average Size [bp]	Size distribution in CV [%]	Conc. [pg/µl]	Molarity [pmol/l]	Co lor
200	1,365	2,874.6	96	485	40.4	1,746.29	6,589.7	

12) C2Om6T

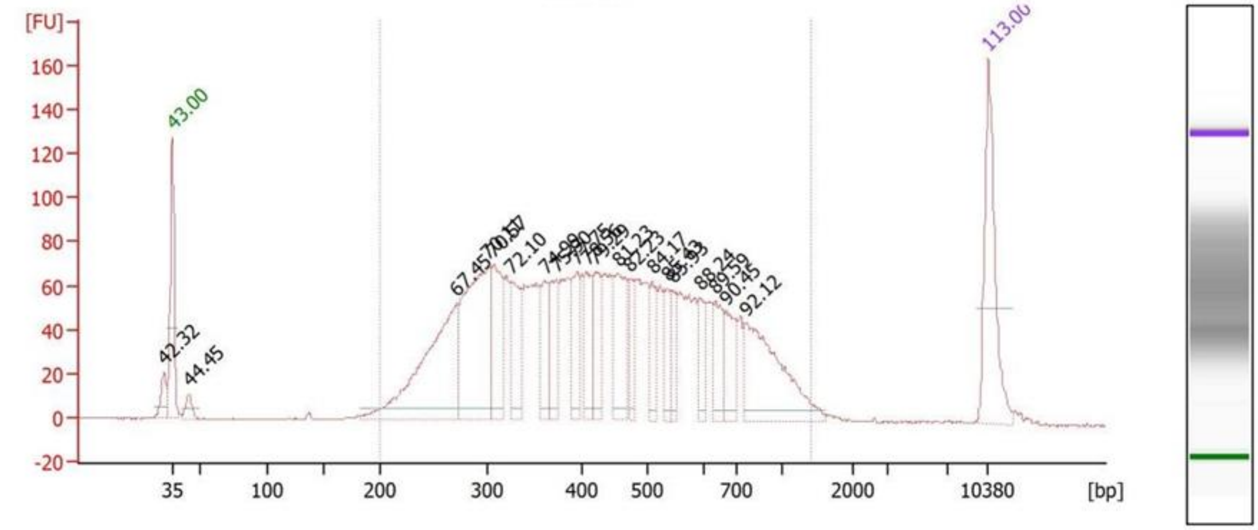

From [bp]	To [bp]	Corr. Area	% of Total	Average Size [bp]	Size distribution in CV [%]	Conc. [pg/µl]	Molarity [pmol/l]	Co lor
200	1,413	2,461.7	96	467	42.1	1,745.85	6,914.1	

13) C2Om12T

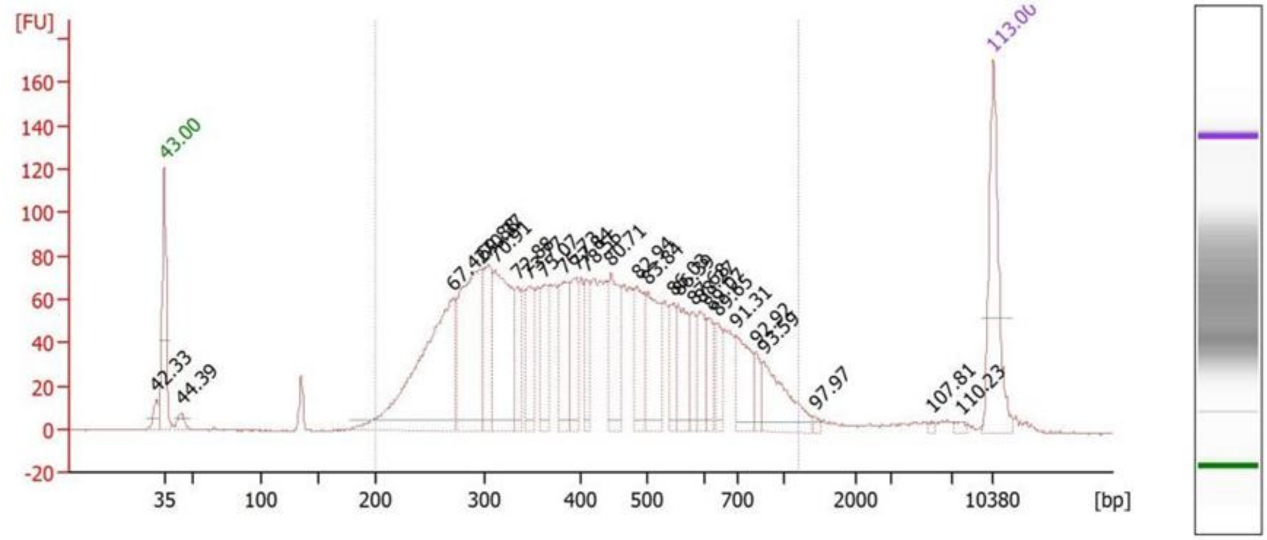

From [bp]	To [bp]	Corr. Area	% of Total	Average Size [bp]	Size distribution in CV [%]	Conc. [pg/µl]	Molarity [pmol/l]	Co lor
200	1,206	2,539.1	94	444	38.7	1,884.80	7,690.7	

14) C2Om24T

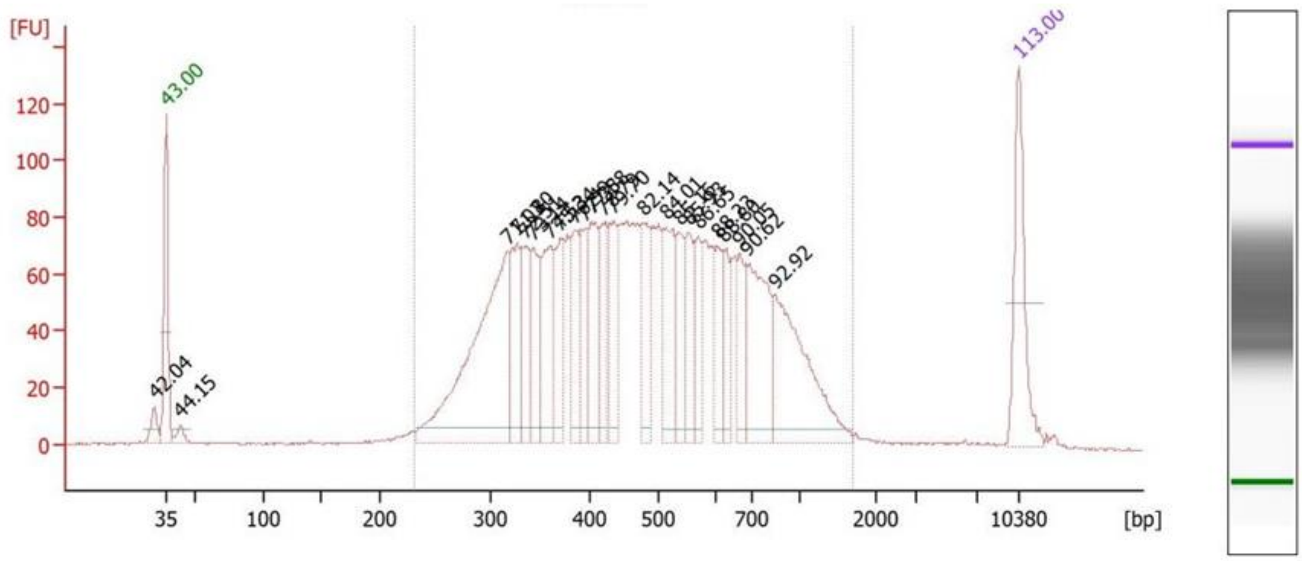

From [bp]	To [bp]	Corr. Area	% of Total	Average Size [bp]	Size distribution in CV [%]	Conc. [pg/µl]	Molarity [pmol/l]	Co lor
231	1,713	2,559.5	95	520	43.4	2,308.57	8,146.3	

15) C3CC6T

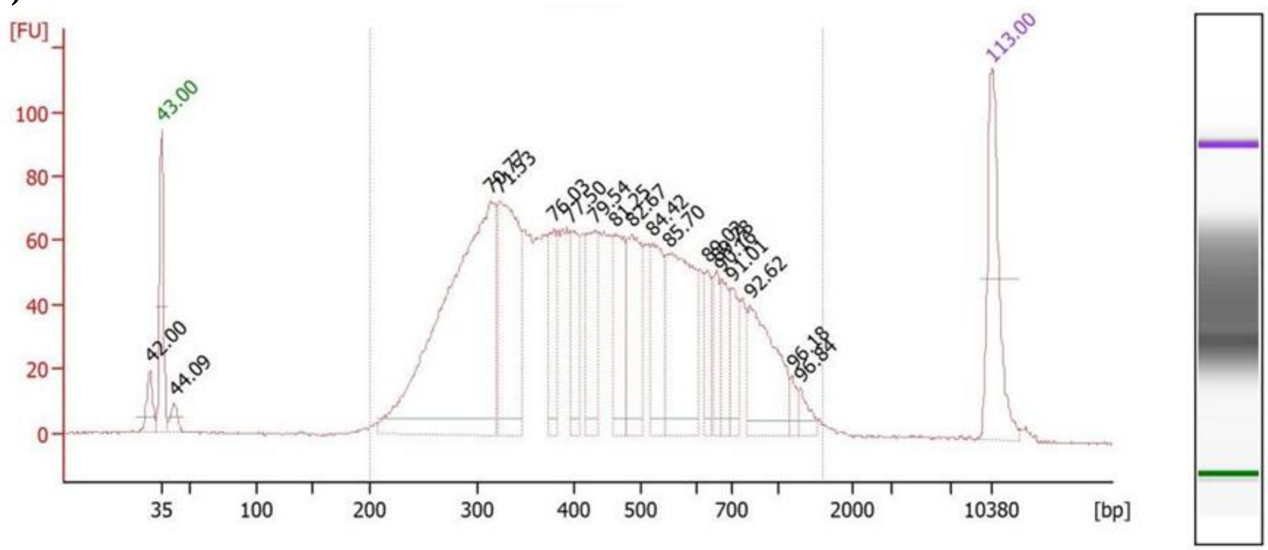

From [bp]	To [bp]	Corr. Area	% of Total	Average Size [bp]	Size distribution in CV [%]	Conc. [pg/µl]	Molarity [pmol/l]	Co lor
200	1,612	2,348.8	96	481	44.4	2,135.71	8,248.9	

16) C3δ6T

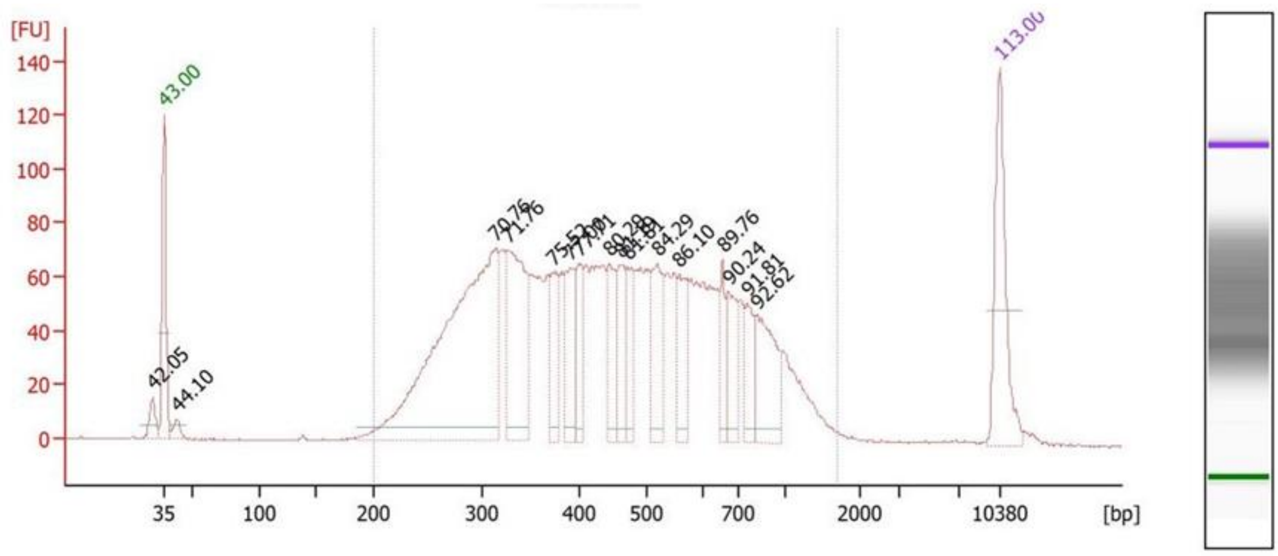

From [bp]	To [bp]	Corr. Area	% of Total	Average Size [bp]	Size distribution in CV [%]	Conc. [pg/µl]	Molarity [pmol/l]	Color
200	1,704	2,471.5	97	493	46.2	1,996.87	7,658.2	

17) C3δ12T

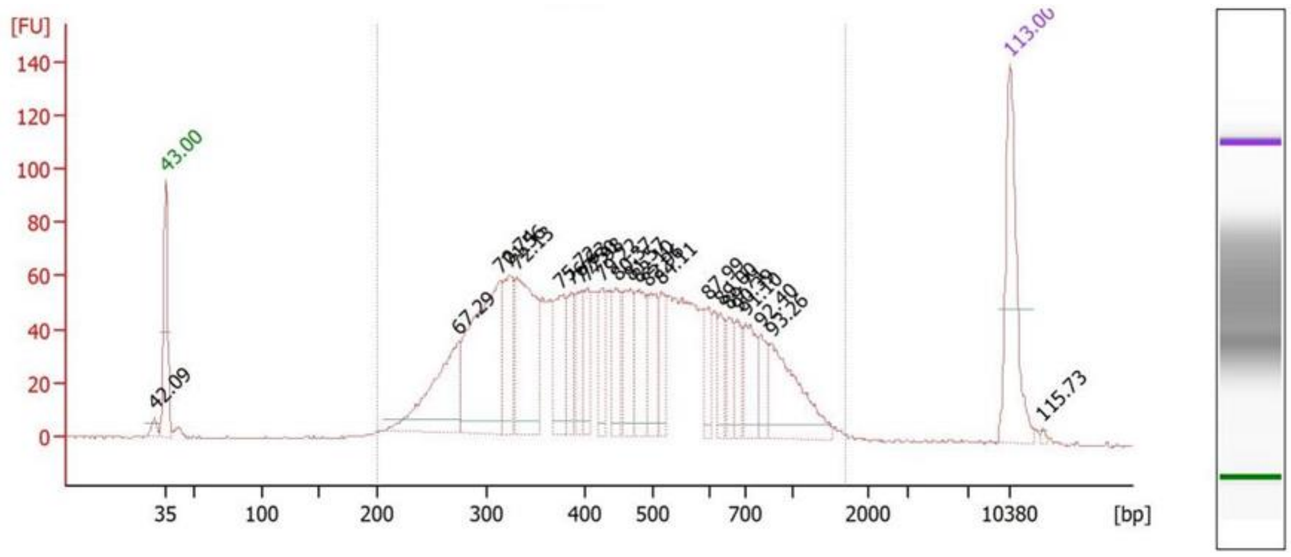

From [bp]	To [bp]	Corr. Area	% of Total	Average Size [bp]	Size distribution in CV [%]	Conc. [pg/µl]	Molarity [pmol/l]	Color
200	1,695	2,035.7	97	493	45.0	1,622.00	6,146.9	

18) C3δ24T

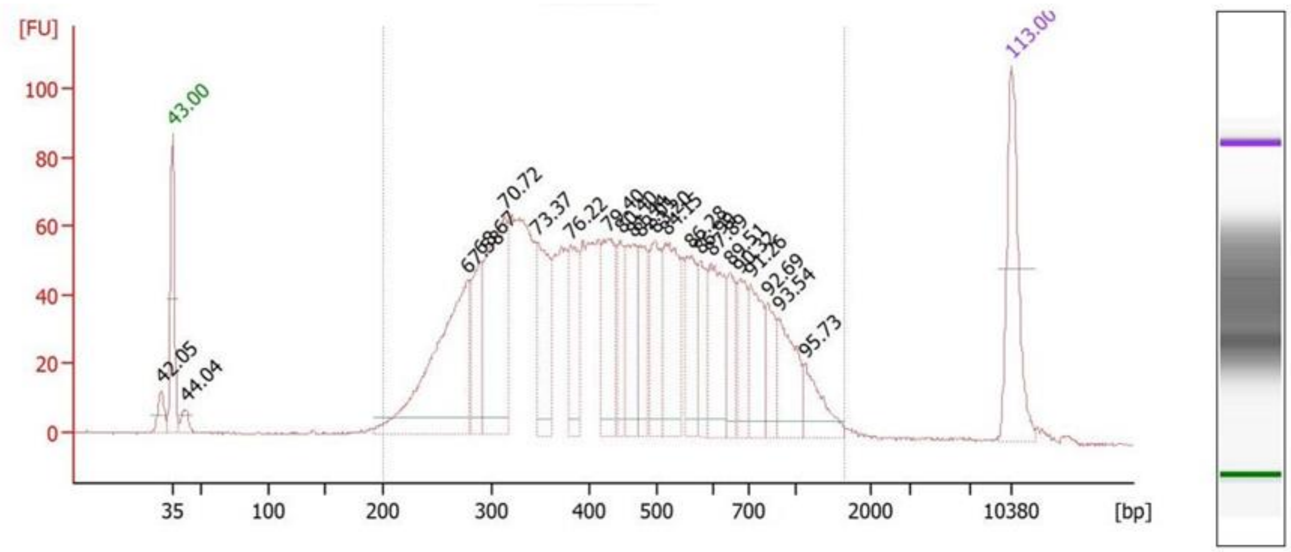

From [bp]	To [bp]	Corr. Area	% of Total	Average Size [bp]	Size distribution in CV [%]	Conc. [pg/µl]	Molarity [pmol/l]	Color
200	1,644	2,127.6	97	487	45.4	2,066.64	7,976.6	

19) C3Om6T

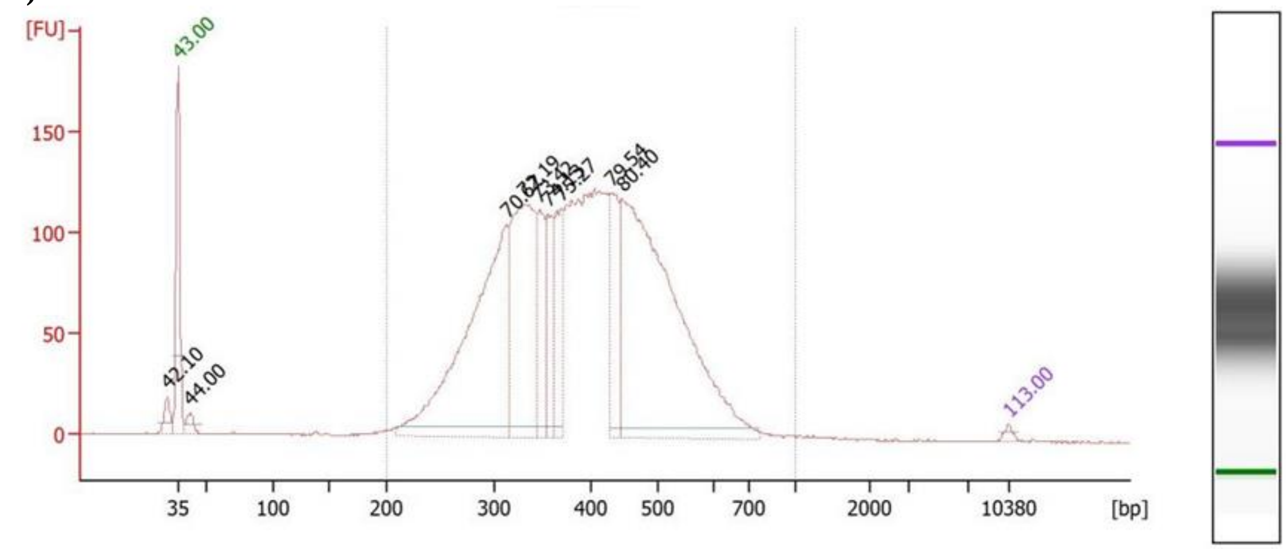

From [bp]	To [bp]	Corr. Area	% of Total	Average Size [bp]	Size distribution in CV [%]	Conc. [pg/µl]	Molarity [pmol/l]	Color
200	1,000	2,931.1	97	401	24.0	50,534.24	206,769.4	

20) C3Om12T

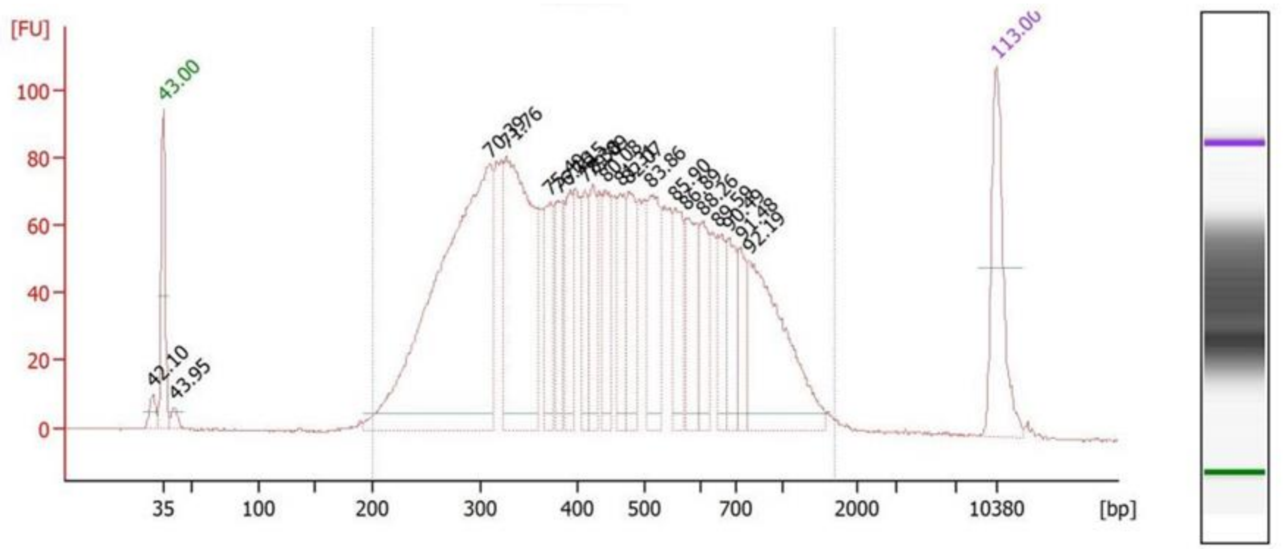

From [bp]	To [bp]	Corr. Area	% of Total	Average Size [bp]	Size distribution in CV [%]	Conc. [pg/µl]	Molarity [pmol/l]	Color
200	1,700	2,729.5	98	485	46.5	2,554.65	9,957.3	

21) C3Om24T

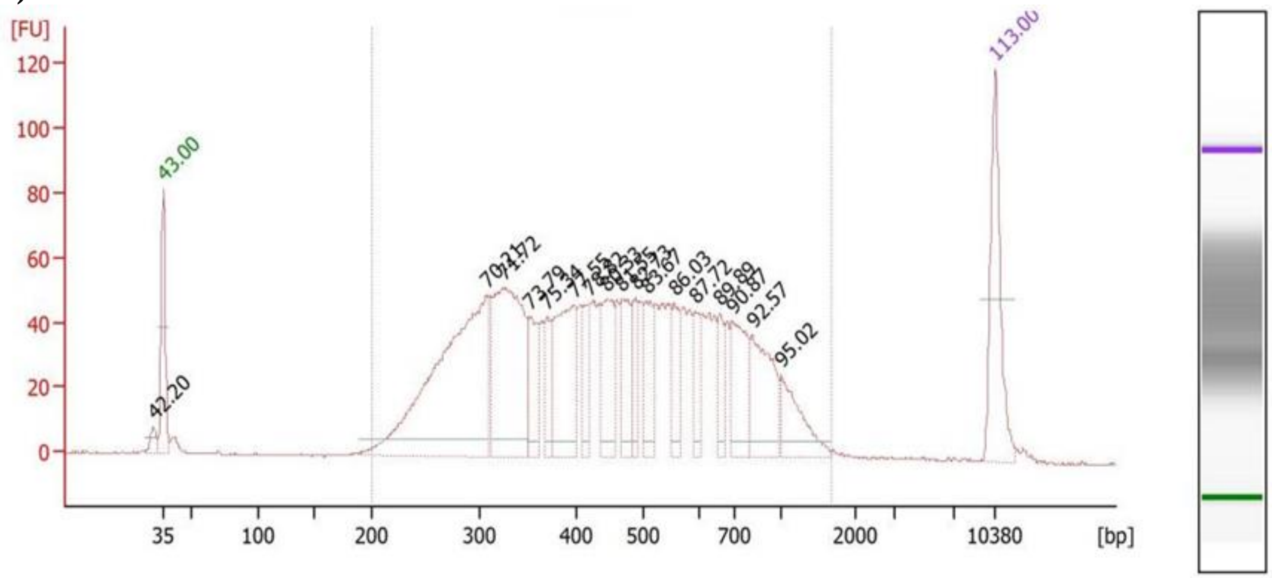

Supplement: Supplementary file 1 [file DataSheet1.pdf]
